# Pigmentosins from *Gibellula* sp. as antibiofilm agents and a new glycosylated asperfuran from *Cordyceps javanica*

**DOI:** 10.3762/bjoc.15.293

**Published:** 2019-12-16

**Authors:** Soleiman E Helaly, Wilawan Kuephadungphan, Patima Phainuphong, Mahmoud A A Ibrahim, Kanoksri Tasanathai, Suchada Mongkolsamrit, Janet Jennifer Luangsa-ard, Souwalak Phongpaichit, Vatcharin Rukachaisirikul, Marc Stadler

**Affiliations:** 1 Microbial Drugs, Helmholtz Centre for Infection Research GmbH (HZI), Inhoffenstraße 7, 38124 Braunschweig, Germany; 2 Department of Chemistry, Faculty of Science, Aswan University, Aswan 81528, Egypt; 3 Department of Microbiology, Faculty of Science, Prince of Songkla University, Songkhla 90112, Thailand; 4 Faculty of Science and Technology, Prince of Naradhiwas University, Khokkhian, Mueang, Narathiwat 96000, Thailand; 5 Computational Chemistry Laboratory, Chemistry Department, Faculty of Science, Minia University, 61519, Egypt; 6 National Centre for Genetic Engineering and Biotechnology (BIOTEC), NSTDA, 113 Thailand Science Park, Phahonyothin Road, Klong Nueng, Klong Luang, Pathum Thani 12120, Thailand; 7 Department of Chemistry, Faculty of Science, Prince of Songkla University, Songkhla 90112, Thailand

**Keywords:** antibiofilm agents, natural products, spider-parasitic fungi

## Abstract

In the course of our exploration of the Thai invertebrate-pathogenic fungi for biologically active metabolites, pigmentosin A (**1**) and a new bis(naphtho-α-pyrone) derivative, pigmentosin B (**2**), were isolated from the spider-associated fungus *Gibellula* sp. Furthermore, a new glycosylated asperfuran **3**, together with one new (**6**) and two known (**4** and **5**) cyclodepsipeptides, was isolated from *Cordyceps javanica*. The pigmentosins **1** and **2** showed to be active against biofilm formation of *Staphylococcus aureus* DSM1104. The lack of toxicity toward the studied microorganism and cell lines of pigmentosin B (**2**), as well as the antimicrobial effect of pigmentosin A (**1**), made them good candidates for further development for use in combination therapy of infections involving biofilm-forming *S. aureus*. The structure elucidation and determination of the absolute configuration were accomplished using a combination of spectroscopy, including 1D and 2D NMR, HRMS, Mosher ester analysis, and comparison of calculated/experimental ECD spectra. A chemotaxonomic investigation of the secondary metabolite profiles using analytical HPLC coupled with diode array detection and mass spectrometry (HPLC–DAD–MS) revealed that the production of pigmentosin B (**2**) was apparently specific for *Gibellula* sp., while the glycoasperfuran **3** was specific for *C. javanica*.

## Introduction

Nosocomial infections are often associated with the presence of *S. aureus*, generally transmitted either by direct contact with carriers or by medical procedures [1]. *S. aureus* is commonly considered as a cause of tissue-associated and medical device-related, in particular orthopedic implant-related infections, since implants coated with proteins facilitate bacterial attachment and biofilm development [1].

In general, bacteria are known to employ different strategies to cope with the presence of antibiotics, of which a biofilm, an aggregate of microorganisms held together within a self-produced matrix of extracellular polymeric substances, plays an important role as a main virulence determinant in staph infections [1–2]. Within a biofilm, bacteria become tolerant toward antibiotics and host immune responses greater than their planktonic (free-floating) cells, leading to an occurrence of reinfection once the antibiotic therapy is terminated [3–5].

In recent years, efforts to find new molecules that can selectively inhibit biofilms have steadily increased, based on the hypothesis that new agents can effectively disrupt biofilm formation and leave target microbes vulnerable to antibiotics [6]. A complementary approach of using a combination of an antibiotic with a biofilm inhibitor appears to be a promising solution to control biofilm-associated pathogens, as based on the evidence that traditional antibiotics were more effective when used in combination with biofilm inhibitors [7]. Since finding an effective strategy to control biofilm formation remains a challenge, the effort to search for an effective antibiofilm agent was herein made.

Invertebrate-pathogenic fungi, in particular the spider-pathogenic fungi, have recently proved to be a promising source of bioactive compounds [8–10]. Thus, during the current study, which is part of a project aiming to discover novel biofilm inhibitors from Thai fungi [11], a number of invertebrate-pathogenic fungi collected from various parts of Thailand were studied for production of bioactive secondary metabolites. Herein, we report on the isolation, structure elucidation, and biological activities of six compounds from *Gibellula* sp. and *Cordyceps javanica*. Furthermore, the species-specific patterns of secondary metabolite production were studied.

## Results and Discussion

### Structure elucidation

**Figure 1:**
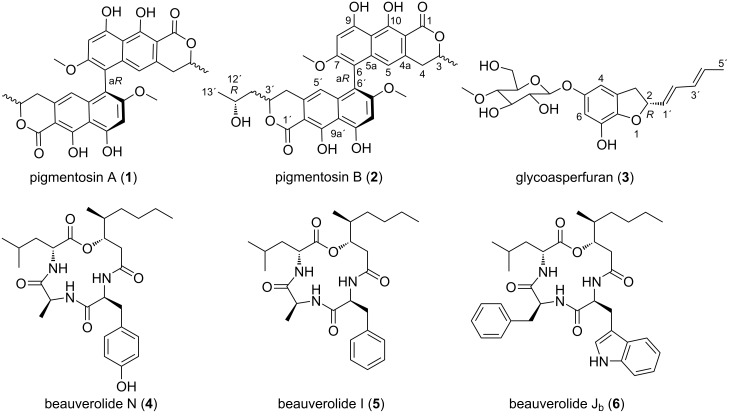
Chemical structures of the isolated compounds **1**–**6**.

*Gibellula* sp. was cultivated in liquid yeast, malt, and glucose (YMG) medium and extracted as described in the Experimental section. The extracts were purified by HPLC to give pigmentosin A (**1**) and pigmentosin B (**2**). Using a similar procedure, compounds **3**–**6** were obtained from the liquid culture of *C. javanica* (Figure 1).

**Figure 2:**
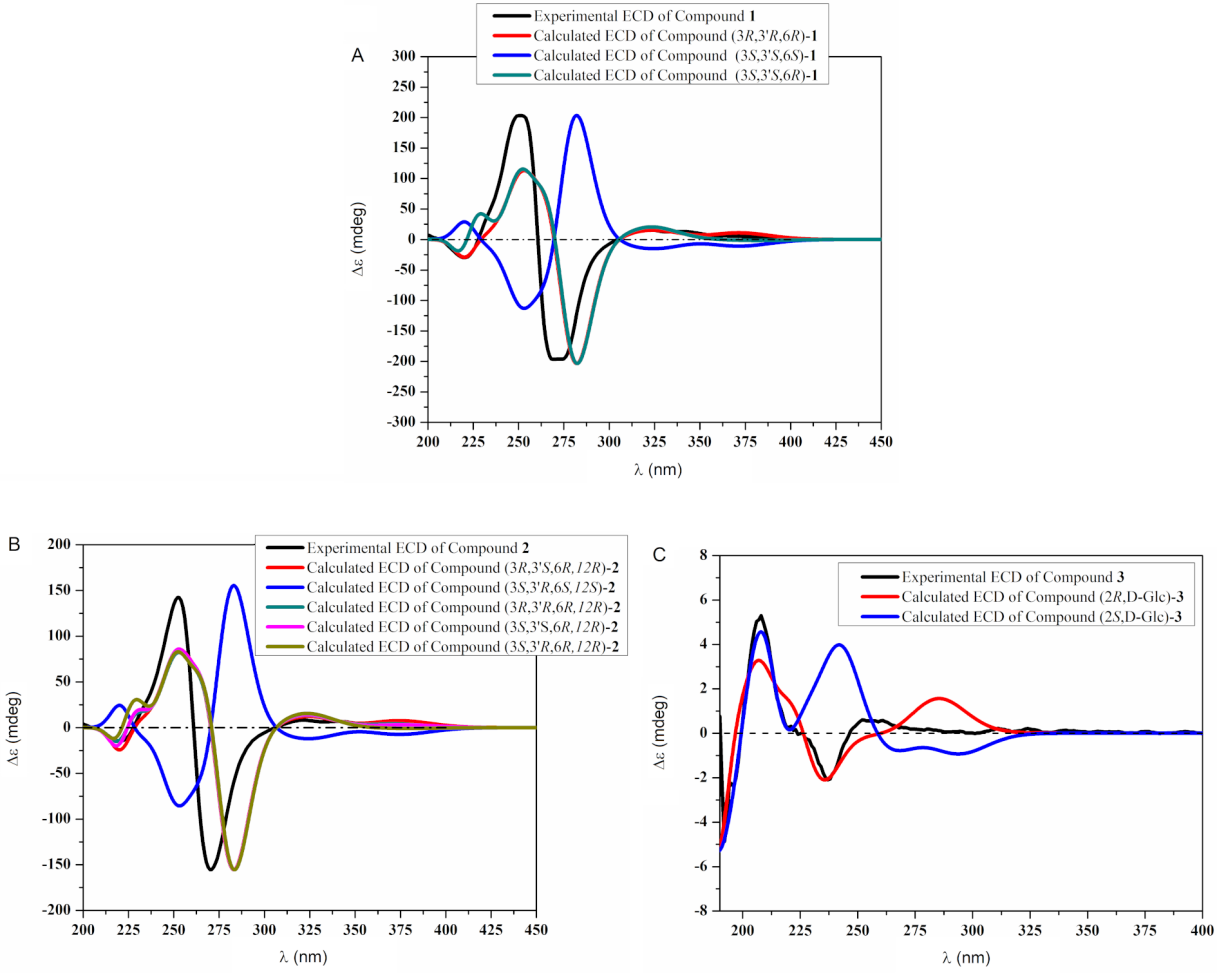
Experimental and TDDFT-calculated ECD spectra of compounds **1** (A), **2** (B), and **3** (C) in MeOH.

Compound **1** was obtained as pale green powder. Its molecular formula was determined as C_30_H_28_O_10_ based on the HRMS data. The presence of only 15 resonances in the ^13^C NMR spectrum suggested a highly symmetric structure. The structure of **1** was then identified to be identical with pigmentosin A, a 3,4-dihydro-α-naphthopyrone dimer with a 7,7′-dimethoxy pattern, by comparing its spectroscopic data with the published data for pigmentosin A [12]. Nevertheless, the chirality of the stereogenic centers C-3/C-3′ as well as the atropisomerism at the 6,6′ axis of pigmentosin A (**1**) were not elucidated previously. Therefore, electronic circular dichroism (ECD) measurements combined with time-dependent density functional theory (TDDFT) calculations of ECD data of compound **1** in MeOH at the B3LYP/6-311+G* level of theory were carried out. The CD spectrum of **1** showed strong Cotton effects: a first negative at 274 nm (Δε −196) and a positive second one at 252 nm (Δε +203), indicating the helicity of the 6-6′ axis as a*R*, according to the exciton chirality method [13]. Furthermore, the TDDFT-ECD calculations were performed on three isomers, namely (3*R*,3′*R*,6*R*)-**1**, (3*S*,3′*S*,6*S*)-**1**, and (3*S*,3′*S*,6*R*)-**1**. The calculated ECD spectrum of (3*R*,3′*R*,6*R*)-**1** reproduced all transitions of the experimental ECD spectrum. In contrast, the (3*S*,3′*S*,6*S*)-**1** compound had a mirror image correlation with the experimental Cotton effects, which indicated that the main Cotton effects around 250 and 270 nm were due to atropisomerism (Figure 2), although the TDDFT-ECD curve of (3*S*,3′*S*,6*R*)-**1** showed a further small positive Cotton effect around 228 nm. Nevertheless, due to the high similarity of both curves, we believe that the calculated ECD data could not distinguish between (3*R*,3′*R*,6*R*)-**1** and (3*S*,3′*S*,6*R*)-**1**. Thus, the atropisomerism at the 6-6′ axis of pigmentosin A (**1**) was determined to be a*R*, while the absolute configuration of the stereogenic centers C-3/C-3′ remains unsolved.

**Figure 3:**
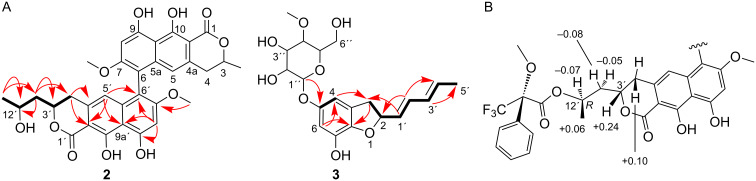
A) Selected COSY (bold bonds) and HMBC (red arrows) correlations for compounds **2** and **3**. B) Partial view of the Mosher ester of pigmentosin B (**2**), showing the shielding effect of the phenyl group of MTPA on the methyl (C-13′), C-3′, and C-4′ positions of **2**. The Δδ*^SR^* values are shown.

Compound **2** was obtained as pale green powder. The molecular formula of **2** was determined as C_32_H_30_O_11_ based on HRMS data. The ^13^C and HSQC NMR data revealed a similarity to compound **1** (Table 1). In addition, the presence of resonances δ_C_ (in ppm) corresponding to two carbonyl carbon atoms at 172.4 (C-1/C-1′), two methyl carbon atoms at 20.8 (C-11′) and 24.1 (C-13′), respectively, two methoxy carbon atoms at 56.5 (C-7/C-7′), two olefinic sp^2^ carbon atoms at 98.7 (C-8/C-8′) and 114.7 (C-5/C-5′), respectively, assigned to four methine units, suggested the presence of a dihydro-α-naphthopyrone moiety in **2**. Comprehensive analysis of the 2D NMR data, including HSQC, COSY, and HMBC, confirmed the structure of **2** as follows: COSY correlations from the methyl H-11 to H-3 and from H-3 to H-4, together with a series of HMBC correlations from H-11 to C-3/C-4, H-4 to C-4a/C-10a/C-5, H-5 to C-4/C-6/C-10a/C-9a, and from H-8 to C9/C-9a/C-6/C-7, confirmed the presence of the dihydro-α-naphthopyrone. The position of the dimethoxy functionality was determined by HMBC correlations from the methoxy functions to C-7/C-7′. Nevertheless, the NMR data revealed differences in the signals of the α-pyrone moieties, suggesting the presence of two asymmetrical dihydro-α-naphthopyrone motifs in **2**. C-3 showed resonances at δ_C_ 79.2 and δ_H_ 4.86, C-3′ at δ_C_ 77.4 and δ_H_ 4.74, C-4 at δ_C_ 33.5 and δ_H_ 2.81/2.95, C-4′ at δ_C_ 35.2 and δ_H_ 2.74/2.87, the two methyl groups C-13′ at δ_C_ 24.1 and δ_H_ 1.17, and C-11 at δ_C_ 20.8 and δ_H_ 1.41. Furthermore, additional signals for an oxygenated methine C-12′ (δ_C_ 63.9 and δ_H_ 3.96) and a methylene group at δ_C_ 44.6 and δ_H_ 1.75/1.97 (C-11′) were observed. Finally, a series of COSY correlations between H-3′, H-11′, H-12′, and H-13′, together with HMBC correlations from H-13′ to C-11′/C-12′ and from H-11′ to C-3′/C-4′/C-12′/C-13′, allowed the assumption that a propan-2-ol moiety was present at C-3′ of the α-pyrone ring on one side of the dimer (Figure 3). Thus, compound **2** was determined to be a new asymmetrical dimer of 3,4-dihydro-α-naphthopyrone and 3-(propan-2-ol)-3,4-dihydro-α-naphthopyrone, a new member of the bis(naphtho-α-pyrone) group [12–13], for which we propose the trivial name pigmentosin B.

**Table 1:** NMR spectroscopic data for pigmentosin B (**2**) and glycoasperfuran (**3**) as well as 4-*O*-methyl-β-ᴅ-glucopyranose for comparison.

**2** (700 MHz, acetone-*d*_6_)			**3** (700 MHz, DMSO-*d*_6_)		

pos.	*δ*_H_, mult., *J* (Hz)	*δ*_C_, type	pos.	*δ*_H_, mult., *J* (Hz)	*δ*_C_, type

1/1′	–	172.4, C	2	5.16, dd, 15.9, 8.2	82.7, CH
3	4.86, br d, 6.9	79.2, CH	3	2.86, dd, 15.7, 7.9 3.26, dd, 15.7, 9.0	36.5, CH_2_
3′	4.74, m	77.4, CH	3a	–	127.6, C
4	2.81, d, obscured 2.95, d, 15.4	33.5, CH_2_	4	6.37, d, 2.1	103.8, CH
4′	2.74, dd, 16.2, 11.1 2.87, br d, 16.2	35.2, CH_2_	5	–	152.1, C
4a/4a′	–	134.7, C	6	6.34, d, 2.1	104.8, CH
5/5′	6.36, s	114.7, CH	7	–	141.0, C
5a/5a′	–	140.3, C	7a	–	141.6, C
6/6′	–	108.6, C	1′	5.74, dd, 15.1, 6.9	129.9, CH
7/7′	–	161.9, C	2′	6.29, dd, 15.3, 10.5	131.9, CH
8/8′	6.82, s	98.7, CH	3′	6.09, dd, 10.7, 14.6	130.6, CH
9/9′	–	159.8, C	4′	5.76, dq, 15.1, 6.9	130.5, CH
9a/9a′	–	111.0, C	5′	1.73, d, 6.5	17.9, CH_3_

10/10′	–	163.0, C	4-*O*-methyl-β-ᴅ-glucopyranose		

10a/10a′	–	100.0, C	1′′	4.60, d, 7.7	101.7, CH
11	1.41, d, 6.9	20.8, CH_3_	2′′	3.15, m	73.5, CH
			3′′	3.36, m	76.2, CH
11′	1.97, m; 1.75, m	44.6, CH_2_	4′′	3.01, t, 9.5	78.9, CH
12′	3.96, m	63.9, CH	5′′	3.23, m	75.5, CH
13′	1.17, d, 6.2	24.1, CH_3_	6′′	3.62, dd, 4.3, 3.1, 11.6 3.50, dd, 6.0, 11.6, 4.7	62.2, CH_2_
7-/7′-OMe	3.76, s	56.5, OCH_3_	4′′-OMe	3.44, s	59.6, CH_3_

**Figure 4:**
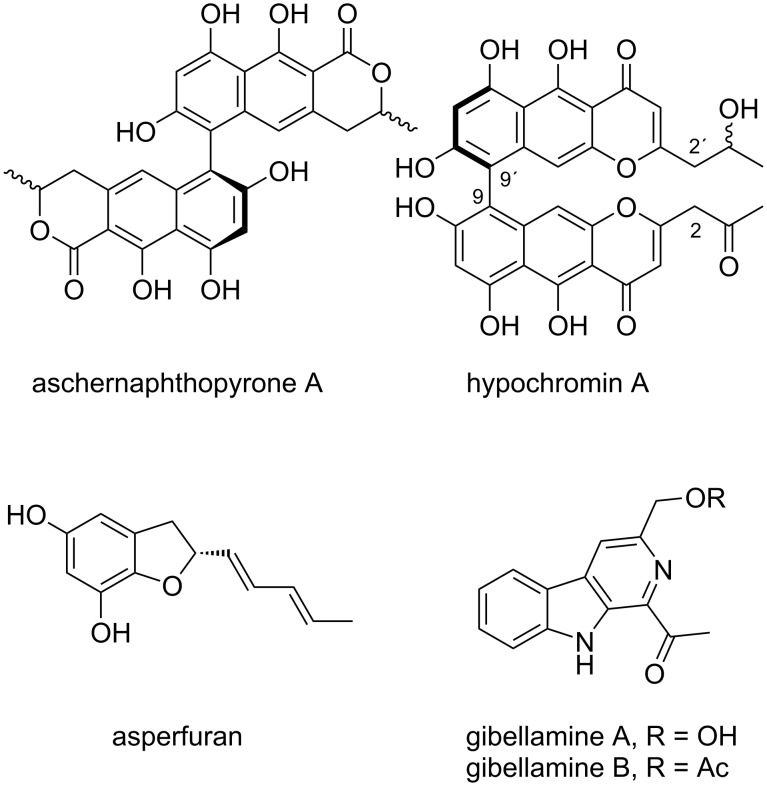
Chemical structures of selected, literature-known compounds that are related to this study.

To determine the absolute configuration at C-12′of pigmentosin B (**2**), Mosher esters of **2** were prepared. The analysis of the Δδ*^SR^* values of the α-methoxy-α-trifluoromethylphenylacetic acid (MTPA) esters were revealed to be negative (−0.07 for H-12′and −0.08/−0.05 for H_2_-11′), while positive Δδ*^SR^* values were observed for H_3_-13′, H-3′, and H_2_-4′ (+0.06, +0.24, and +0.10, respectively). Thus, the absolute configuration at C-12′ was assigned as *R* (Figure 3). The atropisomerism at the 6,6′ axis of pigmentosin B (**2**) was assigned, similarly to pigmentosin A (**1**), by the exciton chirality method. A strong negative first Cotton effect at 271 nm (Δε −155) and a positive second one at 254 nm (Δε +139) indicated the helicity of the 6-6′ axis to be a*R*. This was also confirmed by comparison of the experimental with TDDFT-calculated ECD spectra of (3′*R*,3*S*,6*R*,12*R*)-**2** and (3′*S*,3*R*,6*S*,12*S*)-**2** (Figure 2). The calculated ECD data for (3′*R,*3*S,*6*R,*12*R*)-**2** was in accordance with the experimental ECD data of **2**. TDDFT calculations were also performed on (3′*R*,3*R*,6*R*,12*R*)-**2**, (3′*S*,3*S*,6*R*,12*R*)-**2**, and (3′*S*,3*R*,6*R*,12*R*)-**2** isomers, the corresponding ECD spectra are illustrated in Figure 2. Finally, the calculated ECD spectra were unable to distinguish between (3*R*,3′*S*)-, (3*R*,3′*R*)-, and (3*S*,3′*S*)-isomers. Thus, the configurations at both chiral centers remain unsolved. The CD data of pigmentosins A (**1**) and B (**2**) were similar to those of the related aschernaphthopyrone A [13] and opposite to those of hypochromin A, which possess *S-*configuration with respect to the 9,9′ axis [14] (Figure 4).

The molecular formula of compound **3** was assigned as C_20_H_26_O_8_ based on HRMS data. The ^1^H and ^13^C NMR data (Table 1) showed two characteristic signals that correspond to two olefinic methines (H-4/H-6), and indicated a methylene group (H-3, δ_C/H_ 36.5 and 2.86/3.26) and a highly downfield-shifted oxygenated methine at δ_C_ 82.7/δ_H_ 5.15 (H-2). Furthermore, signals for two conjugated double bonds were observed (δ_H_ 5.74, dd, *J* = 15.1, 6.9 Hz, H-1′; 6.29, dd, *J* = 15.3, 10.5 Hz, H-2′; 6.09, dd, *J* = 14.6, 10.7 Hz, H-3′; 5.76, dq, *J* = 15.1, 6.9 Hz, H-4′), suggesting the presence of a trisubstituted dihydrobenzofuran moiety in **3**. COSY and HMBC correlations allowed the construction of a 1,3-pentadiene moiety (C-1′–C-5′), and the linkage to C-2 of the benzofuran ring was determined by a COSY correlation between H-1′ and H-2, as well as HMBC correlations from H-2′ to C-2 and H-1′ to C-3. Finally, the remaining signals were similar to the reported data for 4-*O*-methyl-β-ᴅ-glucopyranose in our previous reports [8–9]. The HMBC correlation from the anomeric proton H-1′′ to C-6 constructed the glycosidic bond. Thus, compound **3** was determined as a glycosylated derivative of the antifungal asperfuran [15], named glycoasperfuran. The absolute configuration of the sugar moiety was established by comparing the specific rotation of the aqueous layer of its acid hydrolysate ([α]_D_^20^ +30 (*c* 0.02, MeOH)) with that of 4-*O*-methyl-β-ᴅ-glucopyranose ([α]_D_^25^ +80 (*c* 1.3, MeOH)). This was in accordance with our previous reports on akanthopyrones [8]. Finally, the chiral center at C-2 was previously assigned for asperfuran to have *R-*configuration based on the CD spectrum, which showed a negative Cotton effect at 240 nm due to the chirality at C-2, while another asperfuran derivative ((*S*)-4,6-dimethyl-2-vinyldihydrobenzofuran) showed a positive Cotton effect at the same wavelength due to the *S-*configuration at C-2. Since the CD spectrum of glycoasperfuran (**3**) showed a negative Cotton effect at 240 nm, the absolute configuration at C-2 was assigned to be *R*. This was also confirmed by performing TDDFT calculations on the 2*R*/ᴅ-Glc-**3** and 2*S*/ᴅ-Glc-**3** isomers (Figure 2). The calculated ECD spectrum of 2*S*/ᴅ-Glc-**3** showed a main positive Cotton effect at 242 nm, while 2*R*/ᴅ-Glc-**3** had a negative Cotton effect at 237 nm, which was similar to the corresponding experimental ECD spectra. Thus, the absolute configuration of glycoasperfuran (**3**) was confirmed as 2*R*/ᴅ-Glc.

In addition, two known cyclotetradepsipeptides of the beauverolide family, namely beauverolides N (**4**) and I (**5**), and one new beauverolide, J_b_ (**6**), were isolated from *C. javanica* BCC26304. Their structures were identified by comparing HRMS data as well as ^1^H and ^13^C chemical shifts to those reported by Kumza and co-workers [16] for **4** and by Mochizuki and co-workers [17] for **5**. Beauverolide J_b_ (**6**) showed the same molecular formula as beauverolide J_a_ and very similar NMR data [18]. Nevertheless, comprehensive analysis of the 2D NMR data revealed that beauverolide J_b_ (**6**) comprised a leucine moiety instead of isoleucine in beauverolide J_a_ (see NMR data in the Experimental section and Figures S30–S34 in Supporting Information File 1).

### Chemotaxonomic investigation

**Figure 5:**
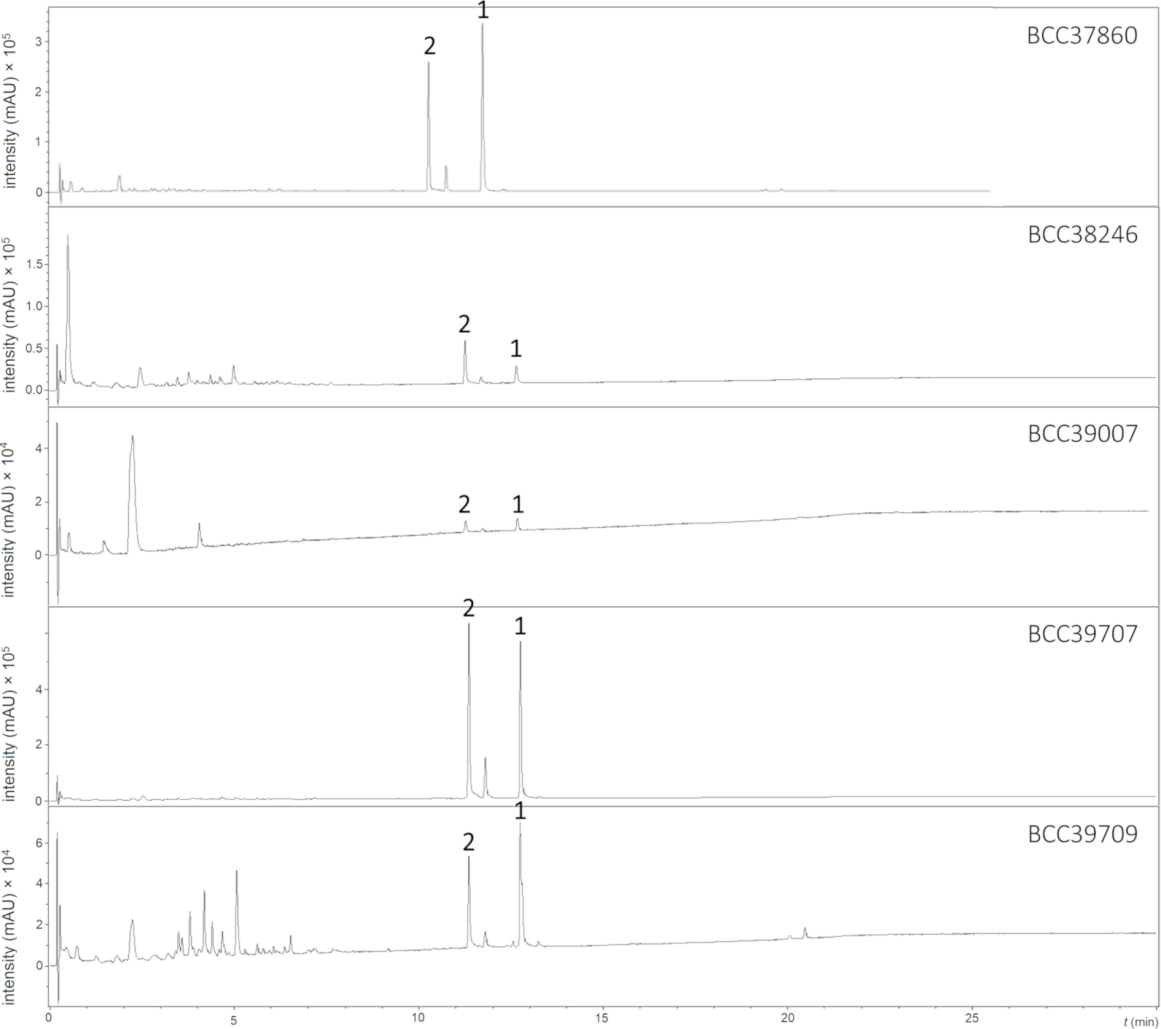
HPLC–UV–vis profiles (200–600 nm) generated from the culture filtrate extracts of several isolates of *Gibellula* sp. Compounds **1** and **2** are pigmentosin A and pigmentosin B.

In order to investigate the distribution patterns of the secondary metabolite production among species of Cordycipitaceae, HPLC–UV–vis profiles of all fungal isolates were generated and compared to each other. This revealed that the individual species possessed unique secondary metabolite profiles. Pigmentosins A (**1**) and B (**2**) were detected in all *Gibellula* strains (Figure 5), but the production rates of each compound varied among strains. Notably, *Gibellula* (class Sordariomycetes) and *Hypotrachyna* (class Lecanoromycetes), from which compound **1** was originally reported, are not phylogenetically close to each other [12,16,18–19], but nevertheless were found to produce the same compound. So far, pigmentosin A (**1**) was reported only from lichenized fungi [20–21], and thus this is the first report of this compound stemming from another group of fungi. Recently, we have reported on the two new β-carboline alkaloid derivatives gibellamines A and B from *Gibellula gamsii*. In the current study, our efforts focused on phylogenetic analysis in order to identify the producers of pigmentosins **1** and **2** as well as glycoasperfuran (**3**), and gibellamines producers were also included in the dataset. Both phylogenetic data and HPLC-based metabolite profiles supported discrimination between these two species, as they were phylogenetically distinct from each other and had individually unique chemotypes.

**Figure 6:**
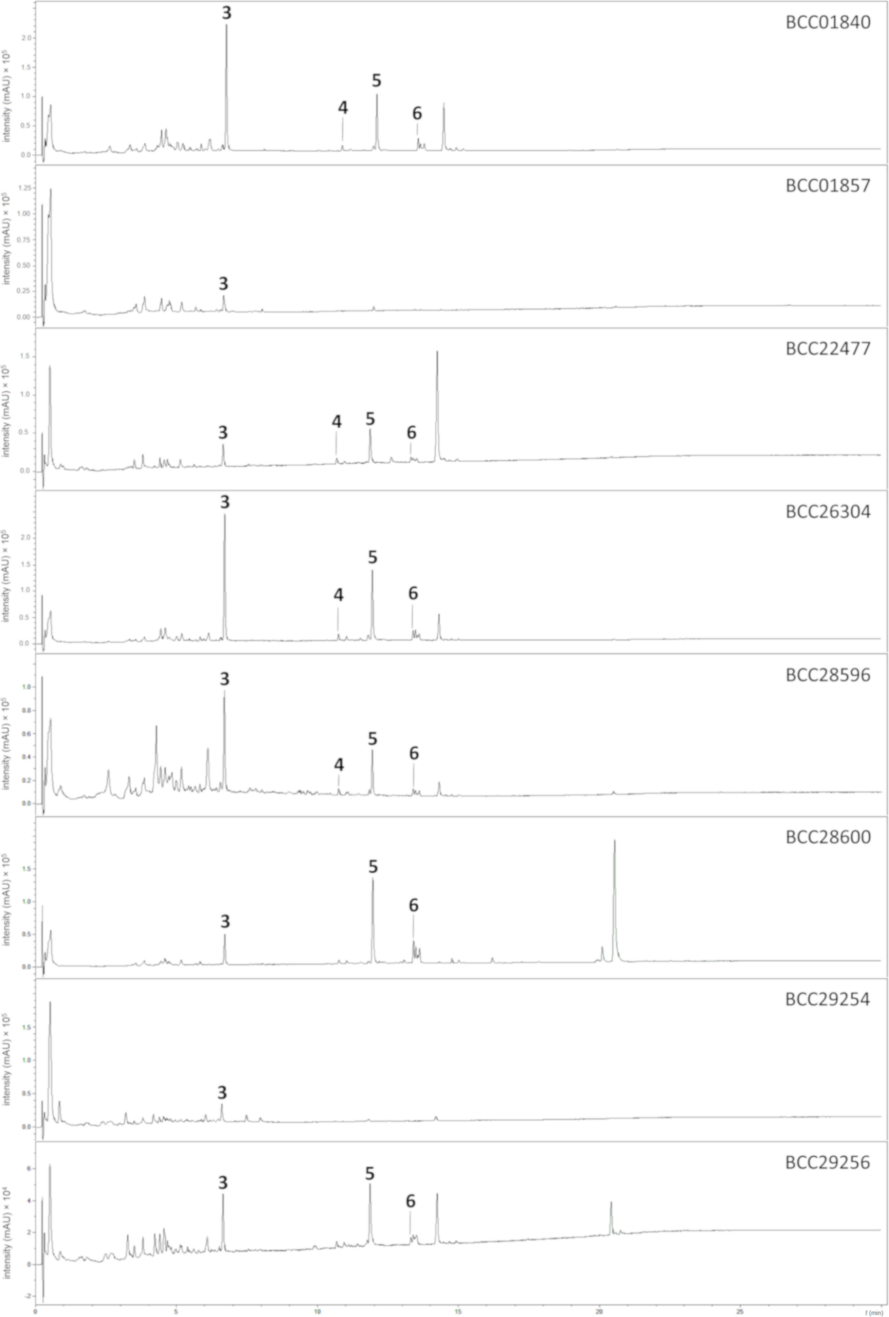
HPLC–UV–vis profiles (200–600 nm) generated from the culture filtrate extracts of several isolates of *C. javanica*. The presence of glycoasperfuran (**3**), beauverolide N (**4**), beauverolide I (**5**), and beauverolide J_b_ (**6**), respectively, is indicated.

The comparison of HPLC–UV–vis profiles showed that in *C. javanica*, glycoasperfuran (**3**), and beauverolides I (**5**) [22] and J_b_ (**6**) were present in all isolates, with the exceptions of the isolates BCC01857 and BCC29254, from which only glycoasperfuran (**3**) could be detected, while beauverolide N (**4**) [15] was produced in trace amounts and only seen in four out of eight isolates (Figure 6). *Beauveria* and *Cordyceps* (*Isaria*) have been found to be phylogenetically close to each other [23], and they produce the same secondary metabolites according to the evidence from Kadlec and co-workers [24], Jegorov and co-workers [25], and Luangsa-ard and co-workers [26]. They unveiled the existences of beauverolides and beauvericin, originally described from *Beauveria*, in *Isaria*-producing *Cordyceps* species*.* Therefore, our results represent proof of finding the cyclotetradepsipeptides beauverolides as common metabolites in *Cordyceps* and *Beauveria*, and glycoasperfuran (**3**) as species-specific metabolite in *C. javanica*.

Recently, Helaly and co-workers [27] described the important role of chemotaxonomy in the modern taxonomy of fungi: 1) secondary metabolite profiles as high-informative data to support morphological and phylogenetic studies, 2) the success of using the polyphasic approach in species delimitation, and 3) the potency of chemotaxonomy in the discovery of numerous new secondary metabolites. In the future, chemotaxonomic studies should therefore be further expanded to other taxa in *Gibellula*, *Cordyceps*, and related genera, since this approach has been mostly restricted only to certain large ascomycete genera.

### Bioactivities

Naphthopyrones are well-known to possess nonselective activities in biological systems and exhibit antimicrobial [28], cytotoxic [29], antimycobacterial [30], and antimalarial [31] effects. Pigmentosin A (**1**) and its new derivative pigmentosin B (**2**) exhibited weak activity against *B. subtilis*, with MIC values of 12.5 and 100 μg/mL, respectively. Notably, **1** also weakly inhibited proliferation of HeLa KB3.1 cells, with an IC_50_ of 17 μg/mL. This compound was reported by Grove and co-workers [32] to be active against *B. subtilis* (MIC 20 μg/mL). Although both compounds showed neither nematicidal activity against *C. elegans* nor antibiofilm activity toward *P. aeruginosa*, they could effectively inhibit the biofilm formation of *S. aureus* with MIC values of 1.9 and 15.6 μg/mL, respectively. On the other hand, they only inhibited growth of *S. aureus* with MIC_90_ values of 31.25 and 250 μg/mL, respectively, while both of them had MBC values higher than 250 μg/mL. Based on the evidence that the cells of *S. aureus* could still grow by more than 80% of the growth control at the lowest concentration where the biofilm formation was interrupted, both pigmentosins **1** and **2** showed antibiofilm effects independently from their antimicrobial activity. Conclusively, even though the mode of action of the pigmentosins remains to be studied, they constitute promising candidates for combination therapy with existing or novel antibiotics.

So far, beauverolides have been found to be potent calmodulin (CaM) inhibitors [33], antiatherosclerotic agents [34], acyl-CoA cholesterol acyltransferase (ACAT) inhibitors [35], insecticides, antimicrobial agents, and immunomodulators [36]. In the current study, beauverolide N (**4**) displayed weak antibiofilm activity against *S. aureus* DSM1104 (MIC 250 μg/mL) and weak cytotoxicity against KB3.1 cells (IC_50_ 16 μg/mL), while beauverolide I (**5**) only exhibited slight inhibition toward the proliferation of the KB3.1 cell line, without either altered or dead cells observed (IC_50_ 20 μg/mL). A number of compounds featuring a dihydrobenzofuran moiety, i.e., the core structure of glycoasperfuran (**3**), has been reported from endophytic [37], invertebrate-pathogenic [38], and marine-derived [39] fungi, or even obtained by total synthesis [40–42], with diverse biological activities [40–41,43–45]. Nevertheless, the new compound, glycoasperfuran (**3**), was devoid of activity in all antimicrobial assays in spite of the fact that the structurally related asperfuran had been reported as a moderately antifungal metabolite [15]. This suggested that the presence of the sugar moiety in glycoasperfuran (**3**) may have led to a loss of activity.

## Conclusion

In the current study, the secondary metabolite profiling using HPLC–DAD–MS led to the isolation of three new compounds (**2**, **3**, and **6**), together with three known metabolites (**1**, **4**, and **5**), as well as species-specific patterns of secondary metabolite production in *Gibellula* sp. and *C. javanica*. Their chemical structures were elucidated based on the interpretation of their NMR and HRMS data. Pigmentosins A and B (**1** and **2**) were isolated from *Gibellula* sp., while glycoasperfuran (**3**) as well as beauverolides N, I, and J_b_ (**4**–**6**) were obtained from *C. javanica*. The absolute configurations of the new compounds pigmentosin B (**2**, partially) and glycoasperfuran (**3**), as well as the atropisomerism in pigmentosin A (**1**), previously unassigned, were determined by a combination of Mosher ester analysis and comparison of the calculated and experimental ECD data. Since pigmentosins A and B (**1** and **2**) were able to significantly inhibit the biofilm formation of *S. aureus*, their bacteriostatic and bactericidal effects were further evaluated. Remarkably, the inhibition toward *S. aureus* and target cell lines was not observed in pigmentosin B (**2**), but only in pigmentosin A (**1**). Nevertheless, pigmentosin A (**1**) displayed anti-*S. aureus* activity independently from its antibiofilm activity. These properties qualified them as promising candidates for alternative antibiofilm agents. We hope that our findings will also help to raise the general scientific interest in invertebrate-pathogenic fungi, and in particular in the taxonomy and secondary metabolism of the spider pathogens.

## Experimental

### General

1D and 2D NMR spectra were recorded on a Bruker Avance III 700 spectrometer with a 5 mm TXI cryoprobe (^1^H NMR: 700 MHz, ^13^C NMR: 175 MHz) and a Bruker Avance III 500 (^1^H NMR: 500 MHz, ^13^C NMR: 125 MHz) spectrometer. Optical rotations were recorded on an Anton Paar MCP 150 polarimeter (sodium D line, Nickel alloy sample cell 100 mm × 3 mm, 0.7 mL). All HPLC–MS analyses were performed on an Agilent 1260 Infinity Systems instrument with a diode array detector and a Waters C_18_ Acquity UPLC BEH column (2.1 mm × 50 mm, 1.7 μm) using the gradient described by Helaly and co-workers [46], combined with ion trap MS (Amazon Speed, Bruker) and HRESIMS–TOF (Maxis, Bruker). Chemicals and solvents were obtained from AppliChem GmbH, Avantor Performance Materials, Carl Roth GmbH & Co. KG, and Merck KGaA in analytical and HPLC grades.

### Fungal material

The invertebrate parasitic fungal specimens were collected from Central and Northeastern Thailand. Their pure cultures were isolated and subsequently deposited at the BIOTEC Culture Collection (BCC), Pathum Thani, Thailand. Two nuclear DNA regions of all isolates including internal transcribed spacer regions of the ribosomal DNA (ITS) and translation elongation factor 1-alpha (EF1-α) were sequenced according to the protocols given by Kuephadungphan and co-workers [10] and Mongkolsamrit and co-workers [47]. The generated sequence data were submitted to GenBank. A list of fungal strains studied and species descriptions are provided in Supporting Information File 1.

### Fermentation and extraction

Submerged fermentation was done as described by Chepkirui and co-workers [48], with minor modifications. Pure cultures were inoculated in YMG liquid medium by cutting seven mycelial plugs (1 cm × 1 cm) from an actively growing colony into a 500 mL Erlenmeyer flask containing 200 mL of the same medium, and incubated at 23 °C on a shaker at 140 rpm. The free glucose content of each fermented broth was tentatively monitored using Bayer Diastix Harnzuckerstreifen. After the glucose was depleted, the incubation was prolonged for half of the time each strain had taken for glucose consumption, and the cultures were then harvested. The fermented broths were separated from the mycelia by vacuum filtration, and were both subsequently extracted according to the procedure described by Phainuphong and co-workers [49]. The fungal mycelia were extracted with acetone, followed by EtOAc instead of MeOH and hexane. Thereafter, the secondary metabolite profiling was carried out on an Agilent 1260 UHPLC Infinity Systems.

Based on a comparison of HPLC profiles between each strain within species, the isolates BCC39707 and BCC26304, representing *Gibellula* sp. and *C. javanica*, respectively, were selected and fermented on a larger scale (4 L) using the procedure described above. After the fermentation was operated in 20 × 500 mL Erlenmeyer flasks containing 200 mL of YMG medium under shaking, the fungal cultures were harvested on day 22, and 5 for *Gibellula* sp. and *C. javanica*. The fermented filtrates from both strains were extracted with 4 L of EtOAc, giving dark brown oily residues, while their mycelia were extracted sequentially with acetone, followed by EtOAc to afford mycelial extracts as brown gum. They were all chemically profiled by HPLC–DAD–MS in order to optimize the chromatographic purification conditions.

### Isolation and structure elucidation of compounds **1**–**6**

The fractionation of the EtOAc extract of *Gibellula* sp. BCC39707 (dissolved in MeOH) was carried out on an Agilent 1100 series HPLC system (Agilent Technologies). The compounds were separated through a reversed-phase C_18_ column (Kromasil, 250 mm × 20 mm, 7 µm, MZ Analysentechnik) using a mixture of deionized water (Milli-Q Millipore, solvent A) and acetonitrile (HPLC grade, solvent B) as eluent, applying a linear gradient of 10–90% solvent B for 50 min, continued to 100% solvent B for 10 min, followed by isocratic conditions of 100% solvent B for 5 min, with a flow rate of 20 mL/min. Thereby, UV detection was performed at 210, 280, and 354 nm. Fractions were collected and pooled according to the observed peaks. The separation yielded two fractions including compound **1** (5.4 mg) and **2** (2.4 mg) at retention times *t*_R_ 43–44 and 37–38 min, respectively. The EtOAc extract of *C. javanica* BCC26304 was also fractionated according to the same manner as the isolation of compounds **1** and **2** with the following gradient: linear gradient of 5–90% solvent B for 50 min, afterwards 100% solvent B for 10 min, thereafter isocratic conditions of 100% solvent B for 5 min to furnish compounds **3** (2.6 mg), **4** (1.3 mg), **5** (9.7 mg), and **6** (2.6 mg) at *t*_R_ 21–23, 35–36, 40–41, and 45–46 min, respectively.

**Pigmentosin A (1)**: Pale green powder; [α]_D_^20^ −128 (*c* 0.2, MeOH); CD (*c* 1 mg/mL, MeOH) λ_max_ (∆ε) 274 (−196), 252 nm (+203); ^1^H NMR (500 MHz, DMSO-*d*_6_) δ 4.70 (m, 2H, H-3/3′), 2.80 and 2.95 (d, *J* = 6.5, 3.4 Hz, 4H, H-4/4′), 6.36 (s, 2H, H-5/5′), 6.82 (s, 2H, H-8/8′), 1.34 (s, 6H, H-11/11′); ^13^C NMR (125 MHz, DMSO-*d*_6_) δ 170.5 (2C, C-1/1′), 76.3 (2CH, C-3/3′), 34.4 (2CH_2_, C-4/4′), 133.9 (2C, C-4a/4a′), 114.7 (2CH, C-5/5′), 138.8 (2C, C-5a/5a′), 109.2 (2C, C-6/6′), 160.9 (2C, C-7/7′), 98.0 (2CH, C-8/8′), 158.7 (2C, C-9/9′), 107.9 (2C, C-9a/9a′), 163.3 (2C, C-10/10′), 99.0 (2C, C10a/10a′), 20.7 (2CH_3_, H-11/11′); LC–MS *m*/*z* (% relative intensity, ion): 547 (100, M^+^ + 1), 1115 (14, 2M^+^ + 1), 545 (100, M^–^ − 1), 1113 (87, 2M^–^ − 1); HRESIMS (*m*/*z*): [M + H]^+^ calcd for C_30_H_29_O_10_, 547.1599; found, 547.1589.

**Pigmentosin B (2)**: Pale green powder; [α]_D_^20^ −17 (*c* 0.1, MeOH); CD (*c* 1 mg/mL, MeOH) λ_max_ (∆ε) 271 (−155), 254 nm (+139); LC–MS *m*/*z* (% relative intensity, ion): 591 (100, M^+^ + 1), 1203 (18, 2M^+^ + 23), 589 (100, M^−^ − 1); HRESIMS (*m*/*z*): [M + H]^+^ calcd for C_32_H_31_O_11_, 591.1861; found, 591.1853; for ^1^H and ^13^C NMR data see Table 1.

**Glycoasperfuran (3)**: Brown powder; [α]_D_^20^ −9 (*c* 0.1, MeOH); CD (*c* 1 mg/mL, MeOH) λ_max_ (∆ε) 237 (−2.1), 209 nm (+4.9); LC–MS *m*/*z* (% relative intensity, ion): 395 (36, M^+^ + 1), 789 (100, 2M^+^ + 23), 393 (35, M^−^ − 1), 787 (84, 2M^−^ − H); HRESIMS (*m*/*z*): [M + H]^+^ calcd for C_20_H_27_O_8_, 395.1700; found, 395.1700; for ^1^H and ^13^C NMR data see Table 1.

**Beauverolide N (4)**: White powder; ^1^H NMR (700 MHz, DMSO-*d*_6_) δ 8.37 (d, *J* = 7.7 Hz, Tyr, NH), 4.02 (q, *J* = 7.7 Hz, Tyr, αCH), 2.82 (m, Tyr, βCH_2_), 6.96 (d, *J* = 8.2 Hz, Tyr, 2CH, *ortho*), 6.64 (d, *J* = 8.6 Hz, Tyr, 2CH, *meta*), 9.24 (s, Tyr, OH), 8.25 (d, *J* = 7.3 Hz, Ala, NH), 3.90 (dt, *J* = 14.1, 6.9 Hz, Ala, αCH), 1.14 (d, *J* = 6.9 Hz, Ala, βCH_3_), 7.19 (d, *J* = 9.0 Hz, Leu, NH), 4.38 (q, *J* = 7.7 Hz, Leu, αCH), 1.44 (dd, *J* = 7.7, 7.3 Hz, Leu, βCH_2_), 1.50 (m, Leu, γCH), 0.85 (d, *J* = 6.9 Hz, Leu, δ_1_CH_3_), 0.88 (d, *J* = 6.5 Hz, Leu, δ_2_CH_3_), 2.32 (dd, *J* = 13.9, 8.6 Hz, CH_2_-2a), 2.42 (dd, *J* = 13.9, 4.5 Hz, CH_2_-2b), 4.83 (ddd, *J* = 10.3, 5.6, 4.3 Hz, CH-3), 2.07 (m, CH-4), 1.02 (m, CH_2_-5a), 1.37 (m, CH_2_-5b), 1.15 (m, CH_2_-6a), 1.28 (m, CH_2_-6b), 1.25 (m, CH_2_-7), 0.86 (t, *J* = 7.3 Hz, CH_3_-8), 0.80 (d, *J* = 6.9 Hz, CH_3_-9); ^13^C NMR (175 MHz, DMSO-*d*_6_) δ 171.0 (Tyr, CO), 56.9 (Tyr, αCH), 34.7 (Tyr, βCH_2_), 127.5 (Tyr, γC), 129.9 (Tyr, 2CH, *ortho*), 114.9 (Tyr, 2CH, *meta*), 155.9 (Tyr, CH, *para*), 170.7 (Ala, CO), 48.3 (Ala, αCH), 15.6 (Ala, βCH_3_), 169.5 (Leu, CO), 51.9 (Leu, αCH), 40.7 (Leu, βCH_2_), 24.3 (Leu, γCH), 21.9 (Leu, δ_1_CH_3_), 22.1 (Leu, δ_2_CH_3_), 170.2 (C-1), 35.4 (CH_2_-2), 75.6 (CH-3), 34.8 (CH-4), 30.5 (CH_2_-5), 28.8 (CH_2_-6), 22.4 (CH_2_-7), 14.0 (CH_3_-8), 15.4 (CH_3_-9); LC–MS *m*/*z* (% relative intensity, ion): 504 (48, M^+^ + 1), 1007 (100, 2M^+^ + 1), 502 (20, M^−^ − 1), 1005 (100, 2M^−^ − 1); HRESIMS (*m*/*z*): [M + H]^+^ calcd for C_27_H_42_N_3_O_6_, 504.3068; found, 504.3072.

**Beauverolide I (5)**: White powder; ^1^H NMR (500 MHz, DMSO-*d*_6_) δ 8.44 (d, *J* = 7.3 Hz, Phe, NH), 4.11 (q, *J* = 7.7 Hz, Phe, αCH), 2.95 (d, *J* = 8.5 Hz, Phe, βCH_2_), 7.20 (m, Phe, 2CH, *ortho*), 7.27 (m, Phe, 2CH, *meta*), 7.20 (m, Phe, CH, *para*), 8.30 (d, *J* = 7.3 Hz, Ala, NH), 3.91 (quin, *J* = 6.9 Hz, Ala, αCH), 1.14 (d, *J* = 6.7 Hz, Ala, βCH_3_), 7.20 (d, *J* = 7.5 Hz, Leu, NH), 4.39 (q, *J* = 7.9 Hz, Leu, αCH), 1.44 (t, *J* = 7.6 Hz, Leu, βCH_2_), 1.50 (m, Leu, γCH), 0.85 (d, *J* = 6.4 Hz, Leu, δ_1_CH_3_), 0.87 (d, *J* = 6.4 Hz, Leu, δ_2_CH_3_), 2.32 (dd, *J* = 14.1, 8.9 Hz, CH_2_-2a), 2.42 (dd, *J* = 14.1, 4.5 Hz, CH_2_-2b), 4.85 (m, CH-3), 2.06 (m, CH-4), 1.01 (m, CH_2_-5a), 1.36 (m, CH_2_-5b), 1.15 (m, CH_2_-6a), 1.24 (m, CH_2_-6b), 1.23 (m, CH_2_-7), 0.85 (t, *J* = 7.2 Hz, CH_3_-8), 0.80 (d, *J* = 6.7 Hz, CH_3_-9); ^13^C NMR (125 MHz, DMSO-*d*_6_) δ 170.8 (Phe, CO), 56.6 (Phe, αCH), 35.5 (Phe, βCH_2_), 137.6 (Phe, γC), 129.1 (Phe, 2CH, *ortho*), 128.2 (Phe, 2CH, *meta*), 126.4 (Phe, CH, *para*), 170.7 (Ala, CO), 48.4 (Ala, αCH), 15.5 (Ala, βCH_3_), 169.5 (Leu, CO), 52.0 (Leu, αCH), 40.7 (Leu, β CH_2_), 24.3 (Leu, γCH), 21.9 (CH_3_, Leu, δ_1_CH_3_), 22.1 (Leu, δ_2_CH_3_), 170.2 (C-1), 35.4 (CH_2_-2), 75.7 (CH-3), 34.8 (CH-4), 30.5 (CH_2_-5), 28.8 (CH_2_-6), 22.4 (CH_2_-7), 13.9 (CH_3_-8), 15.4 (CH_3_-9); LC–MS *m*/*z* (% relative intensity, ion): 488 (39, M^+^ + 1), 975 (100, 2M^+^ + 1), 486 (100, M^−^ − 1), 532 (38, M^−^ + 45), 973 (14, 2M^−^ − 1); HRESIMS (*m*/*z*): [M + H]^+^ calcd for C_27_H_42_N_3_O_5_, 488.3119; found, 488.3121.

**Beauverolide J_b_ (6):** White powder; ^1^H NMR (500 MHz, DMSO-*d*_6_) δ 8.45 (d, *J* = 7.3 Hz, Trp, NH-2), 10.8 (s, Trp, NH-1), 4.20 (dd, *J* = 15.1, 7.7 Hz, Trp, αCH), 3.03 (dd, *J* = 14.6, 8.6 Hz, Trp, β_1_CH_2_), 3.11 (dd, *J* = 14.6, 6.7 Hz, Trp, β_2_CH_2_), 7.08 (1H, s, Trp, CH-2), 7.48 (d, *J* = 7.7 Hz, Trp, CH-4), 7.07 (dd, overlapping, Trp, CH-5), 6.97 (dd, overlapping, Trp, CH-6), 7.36 (d, *J* = 8.2 Hz, Trp, CH-7), 8.20 (d, *J* = 6.9 Hz, Phe, NH), 4.02 (dd, *J* = 7.5, 14.4 Hz, Phe, αCH), 2.85 (dd, *J* = 13.8, 8.6 Hz, Phe, β_1_CH_2_), 3.11 (dd, *J* = 13.3, 6.0 Hz, Tyr, β_2_CH_2_), 6.95 (d, *J* = 6.7 Hz , Phe, 2CH, *ortho*), 7.14 (dd, *J* = 13.7, 6.5 Hz , Phe, 2CH, *meta*), 7.13 (t, *J* = 7.1 Hz, Phe, CH, *para*), 7.38 (d, *J* = 8.6 Hz, Leu, NH), 4.37 (dd, *J* = 7.7, 15.9 Hz, Leu, αCH), 1.44 (m, Leu, βCH_2_), 1.48 (m, Leu, γCH), 0.82 (d, *J* = 6.4 Hz, Leu, δ_1_CH_3_), 0.86 (d, overlapping, Leu, δ_2_-CH_3_), 2.34 (dd, *J* = 14.2, 8.2 Hz, CH_2_-2a), 2.42 (dd, *J* = 14.2, 9.9 Hz, CH_2_-2b), 4.86 (m, CH-3), 2.09 (m, CH-4), 1.02 (m, CH_2_-5a), 1.37 (m, CH_2_-5b), 1.16 (m, CH_2_-6a), 1.23 (m, CH_2_-6b), 1.25 (m, CH_2_-7), 0.84 (t, *J* = 5.9 Hz, CH_3_-8), 0.79 (d, *J* = 6.9 Hz, CH_3_-9); ^13^C NMR (125 MHz, DMSO-*d*_6_) δ 171.6 (Trp, CO), 55.9 (Trp, αCH), 25.7 (Trp, βCH_2_), 123.6 (Trp, CH-2), 109.8 (Trp, C-3), 127.1 (Trp, C-3a), 118.1 (Trp, CH-4), 120.9 (Trp, CH-5), 118.3 (Trp, CH-6), 111.4 (Trp, CH-7), 136.1 (Trp, C-7a), 169.7 (Phe, CO), 54.8 (Phe, αCH), 35.0 (Phe, βCH_2_), 138.8 (Phe, γC), 129.1 (Phe, 2CH, *ortho*), 127.9 (Phe, 2CH, *meta*), 125.9 (Phe, CH, *para*), 169.90 (Leu, CO), 52.1 (Leu, αCH), 40.5 (Leu, βCH_2_), 24.3 (Leu, γCH), 21.8 (Leu, δ_1_CH_3_), 22.1 (CH_3_, Leu, δ_2_CH_3_), 169.92 (C-1), 35.5 (CH_2_-2), 75.7 (CH-3), 34.9 (CH-4), 30.6 (CH_2_-5), 28.7 (CH_2_-6), 22.4 (CH_2_-7), 13.9 (CH_3_-8), 15.4 (CH_3_-9); LC–MS *m*/*z* (% relative intensity, ion): 603 (81, M^+^ + 1), 1205 (94, 2M^+^ + 1), 601 (65, M^−^ − 1), 647 (100, M^−^ + 45), 1203 (24, 2M^−^ − 1); HRESIMS (*m*/*z*): [M + H]^+^ calcd for C_35_H_47_N_4_O_5_, 603.3541; found, 603.3545.

### Preparation of (*S*)- and (*R*)-MTPA esters of pigmentosin B (**2**)

Compound **2** (1.2 mg) was dissolved in deuterated pyridine (1 mL) and transferred into two clean 0.5 mL glass vials. (*R*)-MTPA-Cl (5 μL) was added into one vial to prepare the (*S*)-MTPA ester of **2**, while (*S*)-MTPA-Cl (5 μL) was added into the other vial to prepare the (*R*)-MTPA ester. The reaction was performed at room temperature for 1 h. ^1^H NMR and ^1^H,^1^H-COSY NMR experiments were recorded to obtain the Δδ*^SR^* values. (*S*)-MTPA-**2**: ^1^H NMR (pyridine-*d*_5_) δ 1.36 (3H, H-13′), 5.50 (1H, H-12′), 2.20 (1H, Ha-11′), 1.82 (1H, Hb-11′), 4.90 (1H, H-3′), 2.92 (1H, Ha-4′); (*R*)-MTPA-**2**: ^1^H NMR (pyridine-*d*_5_) δ 1.30 (3H, H-13′), 5.57 (1H, H-12′), 2.28 (1H, Ha-11′), 1.87 (1H, Hb-11′), 4.66 (1H, H-3′), 2.82 (1H, Ha-4′).

### Acid hydrolysis of glycoasperfuran (**3**)

Compound **3** (0.5 mg) was hydrolyzed with 10% aq HCl (1 mL) at 90 °C for 12 h. The reaction mixture was then diluted with H_2_O (2 mL) and extracted with EtOAc (2 × 3 mL). The aqueous layer was concentrated in vacuum to yield **3**. [α]_D_^20^ +30 (*c* 0.02, MeOH).

### Biological assays

To evaluate the biological effects of compounds **1**–**6**, various assays were carried out. The antimicrobial activity of the isolated compounds against *Bacillus subtilis* DSM10, *Escherichia coli* DSM498, *Candida tenuis* MUCL29892, and *Mucor plumbeus* MUCL49355 was determined as described by Kuephadungphan and co-workers [8]. The nematicidal activity against *Caenorhabditis elegans* was investigated using a microtiter plate assay according to Helaly and co-workers [9], while the cytotoxicity was tested against murine fibroblast (L929) and human HeLa (KB3.1) cell lines according to Chepkirui and co-workers [50].

The isolated compounds were also tested for their ability to interfere in the biofilm formation of *Staphylococcus aureus* DSM1104 and *Pseudomonas aeruginosa* PA14 [51]. The biofilm inhibition assay was performed in 96-well microtiter plates using the microtiter dish biofilm formation assay described by O’Toole [52], with minor modifications, as outlined in our recent publications [9,48]. The antibiofilm activity is expressed as MIC values, which is defined as the lowest concentration of substance that prevents biofilm formation of a target microorganism by at least 50%.

Compounds that had shown inhibition of biofilm formation against either *S. aureus* or *P. aeruginosa* were further evaluated for their bacteriostatic and bactericidal activities, as described by Yuyama and co-workers [53], in 8 concentrations, ranging from 1.95–250 µg/mL. The assay was performed with each concentration tested in quadruplicates. The MIC was considered as the lowest concentration where the percentage of inhibition was higher than or equal to 90%. The minimum bactericidal concentration (MBC) of isolated compounds was also determined by transferring an aliquot of 2 µL from all concentrations tested onto nutrient agar (NA) plates, which were then incubated at 30 °C for 24 h. The MBC endpoint was defined as the lowest concentration of the compounds that killed microorganisms, where no visible growth of the microorganism tested was observed on the agar plates. The experimental procedures and the results are given in detail in Supporting Information File 1.

### ECD theoretical calculations

TDDFT-ECD was used to perform theoretical ECD calculations. Conformational searches for the investigated compounds were first performed with a MMFF94S force field and an energy window of 10 kcal/mol using Omega2 software [54–55]. Each resulting conformer was then subjected to geometrical optimization and vibrational frequency calculation at the B3LYP/6-31+G* level of theory using the Gaussian 09 software [56]. Based on the optimized geometries, TDDFT calculations were finally carried out at the B3LYP/6-311+G* level of theory, and the first 50 excitation states were considered. To consider the solvent effect, the polarizable continuum model (PCM) for methanol was applied. ECD spectra were obtained using SpecDis 1.71 [57–58] and averaged using Boltzmann factors evaluated at 293 K. In the calculated/experimental ECD comparison, wavelength shifts and intensity scaling were applied.

## Supporting Information

File 1 LC–MS and NMR data of compounds **1**–**6**, experimental procedures and detailed results for bioassays, as well as species identification of the pigmentosin and glycoasperfuran producers.
