# Supplementary material for: Pigmentosins from Gibellula sp. as antibiofilm agents and a new glycosylated asperfuran from Cordyceps javanica
Source: Beilstein J Org Chem. 2019 Dec 16;15:2968–81. doi: 10.3762/bjoc.15.293 (PMC6941404; doi:10.3762/bjoc.15.293)
Supplement: File 1 — LC–MS and NMR data of compounds 1–6, experimental procedures and detailed results for bioassays, as well as species identification of the pigmentosin and glycoasperfuran producers. [file Beilstein_J_Org_Chem-15-2968-s001.pdf]

BEILSTEIN JOURNAL OF ORGANIC CHEMISTRY

Supporting Information

for

Pigmentosins from *Gibellula* sp. as antibiofilm agents and a new glycosylated asperfuran from *Cordyceps javanica*

Soleiman E. Helaly, Wilawan Kuephadungphan, Patima Phainuphong, Mahmoud A. A. Ibrahim, Kanoksri Tasanathai, Suchada Mongkolsamrit, Janet Jennifer Luangsa-ard, Souwalak Phongpaichit, Vatcharin Rukachaisirikul and Marc Stadler

Beilstein J. Org. Chem. **2019,** *15,* 2968–2981. doi:10.3762/bjoc.15.293

LC–MS and NMR data of compounds 1–6, experimental procedures and detailed results for bioassays, as well as species identification of the pigmentosin and glycoasperfuran producers

License and Terms: This is a supporting information file under the terms of the Creative Commons Attribution License (https://creativecommons.org/licenses/by/4.0). Please note that the reuse, redistribution and reproduction in particular requires that the authors and source are credited.
The license is subject to the *Beilstein Journal of Organic Chemistry* terms and conditions: (https://www.beilstein-journals.org/bjoc)

Contents | **Page**

1. *Species identification*		
Table S1	List of taxa used in the current phylogenetic study	S2
Fig. S1	RAxML tree based on the concatenated two gene dataset (ITS and EF1-α)	S4
2. *Biological activity screening of compounds **1**–**6***		S5
Table S2	Antimicrobial, cytotoxic and nematicidal activities of compounds **1**–**6**	S7
Table S3	Anti-biofilm activity of compounds **1**–**6**, bacteriostatic and bactericidal activities of active compounds	S7
3. *HRMS and NMR spectral data for compounds **1–6***		S8
4. *References*		S24

Species identification

The species identification of the studied isolates relied solely on phylogenetic analysis inferred from the combined sequence datasets of ITS and EF1-α. The sequence data (Table S1) of these two loci were generated according to the protocol described by Kuephadungphan et al.[1] The phylogenetic tree construction was subsequently conducted in RAxML 7.2.8 plugin on Geneious® 7.1.9[2–3]using GTR+G as substitution model. Relative support for the branches was obtained from bootstrap analysis with 1,000 replicates. The RAxML tree (Fig. S1) revealed thatthe studied isolates were distributed in strong supported *Cordyceps* and *Gibellula* clades. Based on the close relationship between pigmentosin producers and the type strain of *C. javanica*, they could be undoubtedly identified as*C. javanica* while all glycoasperfuran-producing isolates were placed together in a 100% BS supported clade without any related taxa, they were then considered as unidentified *Gibellula* species.

Table S1 List of taxa used in the current phylogenetic study. The type strains are indicated with a superscript T (^{T})and the studied isolates are in bold.

Species	Strain number	GenBank accession no.	
		ITS	EF1-α
Akanthomyces aculeatus	HUA186145^{T}		MF416465
Akanthomyces aculeatus	HUA772	KC519371	KC519366
Akanthomyces farinosa	CBS541.81		MF416498
Akanthomyces tuberculatus	HUA186131		MF416466
Akanthomyces sabanensis	ANDES-F1014	KC633245	KC875221
Akanthomyces sabanensis	ANDES-F1024^{T}	KC633232	KC633266
Beauveria bassiana	ARSEF 1564^{T}	HQ880761	HQ880974
Beauveria bassiana	ARSEF7518	HQ880762	HQ880975
Beauveria diapheromeriphila	QCNE186272^{T}	JQ958599	JQ958610
Beauveria diapheromeriphila	QCNE186714	JQ958603	JQ958611
Blackwellomyces cardinalis	OSC93609^{T}		DQ522325
Blackwellomyces cardinalis	OSC93610		EF469059
Cordyceps amoenerosea	CBS107.73^{T}	AY624168	MF416494
Cordyceps amoenerosea	CBS729.73	AY624169	MF416495
Cordyceps cateniannulata	CBS152.83^{T}	AY624172	JQ425687
Cordyceps cateniobliqua	CBS153.83^{T}	AY624173	JQ425688
Cordyceps chiangdaoensis	TBRC7274^{T}	KT261393	KT261403
Cordyceps cicadae	ARSEF7260	HQ880826	HQ881017
Cordyceps cicadae	RCEFHP090724-31		MF416496
Cordyceps coleopterorum	CBS110.73^{T}	AY624177	JF416028
Cordyceps farinosa	CBS111113^{T}	AY624181	MF416499
Cordyceps fumosorosea	CBS107.10^{T}	AY624184	MF416502
Cordyceps fumosorosea	CBS244.31		MF416503
Cordyceps ghanensis	CBS105.73^{T}	AY624185	
Cordyceps javanica	CBS134.22^{T}	AY624186	MF416504
Cordyceps javanica	TBRC7259	MF140745	MF140831
Cordyceps javanica	TBRC7260	MF140744	MF140830
Cordyceps javanica	**BCC01840**	**MH532892**	**MH521904**
Cordyceps javanica	**BCC01857**	**MH532893**	
Cordyceps javanica	**BCC22477**	**MH532842**	
Cordyceps javanica	**BCC26304**	**MH532851**	**MH521903**
Cordyceps javanica	**BCC28596**	**MH532854**	
Cordyceps javanica	**BCC28600**	**MH532855**	
Cordyceps javanica	**BCC29254**	**MH532856**	
Cordyceps javanica	**BCC29256**	**MH532857**	
Cordyceps kintrischica	ARSEF7218^{T}	EU553278	GU734751

Table S1 (Cont.) List of taxa used in the current phylogenetic study. The type strains are indicated with a superscript T (^{T}) and the studied isolates are in bold.

Species	Strain number	GenBank accession no.	
		ITS	EF1-α
Cordyceps kintrischica	ARSEF8058	GU734764	GU734750
Cordyceps militaris	ARSEF5050	HQ880829	HQ881020
Cordyceps militaris	OSC93623^{T}		DQ522332
Cordyceps morakotii	TBRC7276^{T}	KT261390	KT261400
Cordyceps oncoperae	AFSEF4358^{T}		EF468785
Cordyceps tenuipes	ARSEF5135^{T}	AY624196	JF416020
Cordyceps tenuipes	ARSEF4096	HQ880827	HQ881018
Engyodontium aranearum	CBS309.85	AJ292391	DQ522341
Engyodontium aranearum	CBS658.80	LC092897	
Gibellula clavulifera var. alba	ARSEF1915^{T}	JN049837	DQ522360
Gibellula gamsii	BCC27968^{T}	MH152529	MH152560
Gibellula gamsii	BCC27970	MH152530	MH152561
Gibellula leiopus	BCC16025		MF416492
Gibellula pulchra	NHJ10808		EU369018
Gibellula sp.	NHJ10788		EU369019
***Gibellula* sp.**	**BCC37860**	**MH532871**	
***Gibellula* sp.**	**BCC38246**	**MH532872**	**MH521893**
***Gibellula* sp.**	**BCC39007**	**MH532873**	
***Gibellula* sp.**	**BCC39707**	**MH532875**	**MH521894**
***Gibellula* sp.**	**BCC39709**	**MH532876**	
Hevansia arachnophila	NHJ10469		EU369008
Hevansia cinerea	BCC02191	GQ250000	GQ250029
Hevansia cinerea	BCC47913	JX192716	JX192812
Hevansia novoguineensis	CBS610.80^{T}	SUBMIT	
Hevansia novoguineensis	NHJ11923		EU369013
Hevansia websteri	BCC23860	GQ250009	GQ250030

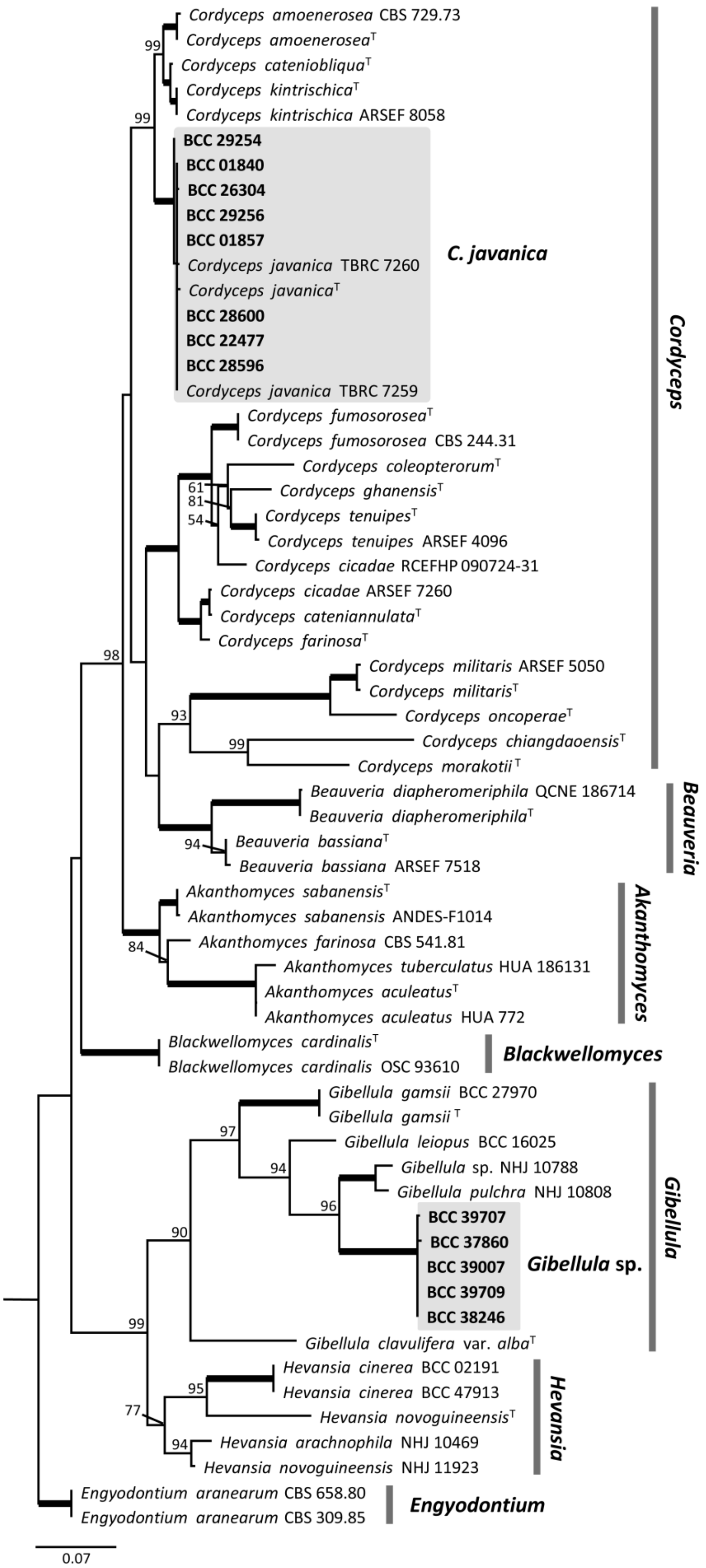

Figure S1 RAxML tree based on the concatenated two gene dataset (ITS and EF1-α) showing the relationship among *Gibellula*, *Cordyceps* and related genera. Bootstrap proportions (BS) ≥50% are provided above corresponding nodes; nodes with a BS of 100% are shown as thick lines. The ex-type strains are marked with a superscript T (^{T}) and the isolates encountered in this study are highlighted.

Biological activity screening of compounds 1–6

Antimicrobial activity assay

The minimum inhibitory concentration (MIC) of compounds **1**–**6** was determined using the broth microdilution method according to Kuephadungphan et al.[4] against *B. subtilis* DSM10, *E. coli* DSM498, *C. tenuis* MUCL29892 and *M. plumbeus* MUCL49355. Stock suspension of each bacterium and yeast (100 µL) was transferred to 100 mL of EBS medium and YM medium, respectively. Suspensions of *B. subtilis* and *C. tenuis* were incubated on a rotary shaker at 30 °C for 18–24 h while *E. coli* was grown at 37 °C for 24 h. After incubation, the suspension was adjusted to a concentration of 6.7×10^5 cells/mL using a hemacytometer. The spore suspension of *M. plumbeus* was prepared at a concentration of 6.7×10^5 conidia/mL using YM medium. The determination of MIC was performed in a 96-well microtiter plate. The compounds dissolved in methanol at a concentration of 4.5 mg/mL (20 µL) were transferred to the first row of the plate. Standard antibiotics including ciprofloxacin (bacteria) and nystatin (yeast and fungus), and methanol were used as positive and negative controls, respectively. Inoculum suspension (280 µL) was added to the first row containing compounds and 150 µL were added to the rest. The solutions were then serially 2-fold diluted to 8 concentrations ranging from 2.34–300 µg/mL. Plates were incubated at 30 °C on a microplate-vibrating shaker for 24 h for bacteria and 48 h for yeast and filamentous fungus. After incubation the lowest concentration of each compound at which no visible growth was observed and recorded as the MIC.

Nematicidal activity assay

The nematicidal activity of the new compounds against *C. elegans* was performed by a microwell plate assay reported by Stadler et al.[5] with slightly modification. The modified protocol was reported by Helaly et al.[6] The studied compounds were dissolved with methanol to a concentration of 100 mg/mL and subsequently diluted 1:100 with sterile distilled water. The free-living nematode, *C. elegans* was monoxenically cultured on nematodes agar (soy peptone 2 g, NaCl 1 g, agar 20 g, 1,000 mL of distilled water, after autoclaving, the following ingredients were added: cholesterol (1 mg/mL EtOH) 0.5 mL, 1 M $CaCl_2$ 1 mL, 1 M $MgSO_4$ 1 mL, 40 mM potassium phosphate buffer 12.5 mL, pH 6.8) with living *E. coli* DSM498, at 20 °C in the dark for a week. After incubation, adult nematodes were suspended in sterile distilled water and transferred to a sterile tube. Finally, the nematodes were counted using a Malassez counting chamber and further adjusted to obtain a concentration of 500 nematodes/mL with sterile distilled water. Nematicidal activity screening was performed in a 24-microwell plate (total volume 1 mL/well) and 4 concentrations of 100, 50, 20 and 10 µg/mL of each compound were tested. Standard nematicide, ivermectin and 1% MeOH were used as positive inhibitory control and solvent control, respectively. The plate was incubated at 20 °C in the dark and nematicidal activity was recorded after 18 h of incubation. The nematodes were counted under a stereo microscope. The living nematodes could be recognized by their snake-like movements while the dead ones appeared to be straight.

Cytotoxicity assay

The isolated compounds were tested for cytotoxic activity using the MTT (3-(4,5-dimethylthiayol-2-yl)-2,5-diphenyltetrayolium bromide) method in 96-well microtiter plates following the procedure previously described by Chepkirui et al.[7] against the cervix carcinoma cell line KB3.1 and the established mouse fibroblast cell line L929. The assay was kindly conducted by Wera Collisi, Department of Microbial Drugs, Helmholtz Centre of Infection Research, Germany. The tested cell lines were cultured in EBM-2 (Lonza) supplemented with 10% fetal bovine serum (Gibco) under 10% CO_2 at 37 °C. A 60 µL of serial dilutions from an initial stock (1 mg/mL) of the test compounds in MeOH was added to 120 µL aliquots of a cell suspension (50,000 cells/mL) in 96-well microplates. After incubation for five days, 20 µL of MTT in phosphate buffered saline (PBS) were added to give a final concentration of 0.5 mg/mL. The plates were further incubated for two hours. The precipitate

of formazan crystals was centrifuged and the supernatant was then discarded. Each well was washed with 100 µL of PBS and 100 µL of isopropanol containing 0.4% hydrochloric acid were subsequently added in order to get the residual dissolved. The plates were gently shaken for 20 min before the absorbance at 590 nm was measured using an ELISA microplate reader (Victor). The concentration, at which the growth of cells was inhibited to 50% of the control (IC_{50}), was obtained from the dose-response curves. Methanol was used as negative control while epothilone A and B were used as positive controls for L929 and KB3.1 cell lines, respectively.

Anti-biofilm activity assay

The determination of the ability of isolated compounds to prevent biofilm formation of *S. aureus* DSM1104 (ATCC25923) and *P. aeruginosa* PA14[8] was performed using the microtiter dish biofilm formation assay described by O'Toole[9] with minor modification provided in our recent publications[1, 6, 10–14]. The biofilm forming strains were enriched overnight in CASO medium with and without 4% glucose for *S. aureus* and *P. aeruginosa*, respectively. After incubation at 37 °C under shaking condition, the overnight cultures were adjusted to equal the turbidity of 0.5 McFarland standard using the assay medium. The assay was carried out in 96-well tissue cultured-treated microplates (TPP®, Germany) for *S. aureus* and non-treated plates (Falcon®MicroTestTM, USA) for *P. aeruginosa*, in which 10 µL of each compound (0.5 mg/mL) in six replicates were mixed together with 140 µL of bacterial suspension. Methanol and CASO medium with and without 4% glucose were used as negative controls and tetracycline (100 µg/mL) as positive control. Plates were covered with a sterile adhesive porous paper (Kisker Biotech GmbH, Steinfurt, Germany) and incubated at 37 °C for 24 h. After incubation, bacterial biofilms were stained with 0.1% crystal violet solution (Sigma-Aldrich) following the protocol of O'Toole.[9] Then, the biofilm was subsequently quantified using a microplate reader at 550 nm.[12] In the current study, the antibiofilm activity was expressed as MIC value which was defined as the lowest concentration of substance that prevents the biofilm formation of a target microorganism by at least 50%.

Bacteriostatic and bactericidal activity assay

The compounds that possessed the biofilm activity against either *S. aureus* DSM1104 or *P. aeruginosa* PA14 were further evaluated for their bacteriostatic and bactericidal activities. The assay was performed in the Bioscreen 100-well Honeycomb plate according to the protocol described by Yuyama et al.[13] where the MIC values were determined. The bacterial inoculum was prepared by growing *S. aureus* DSM1104 in Luria-Bertani broth (LB) for 24 h at 30 °C and then adjusting to 0.5 McFarland turbidity standard. The 0.5 McFarland bacterial suspension (296.25 µL) was transferred to the well containing 3.75 µL of each compound (20 mg/mL) and was subsequently two-fold diluted to give 8 concentrations ranging from 250–1.95 µg/mL. Since compound **2** and the remaining five compounds could completely be dissolved in MeOH and dimethyl sulfoxide (DMSO), respectively, MeOH and DMSO were used as the solvent controls. The assay was performed with each concentration tested in quadruplicate. The bacterial growth was monitored on the Bioscreen-C automated growth curve analysis system (Oy Growth Curves AB Ltd, Helsinki, Finland) for 24 h with the optical density at 600 nm measured at 15-min intervals. The MIC of each compound tested was read at 24 h of incubation and was considered as the lowest concentration where the percentage of inhibition was higher than or equal to 90%. Herein, the minimum bactericidal concentration (MBC) of isolated compounds was also determined by transferring an aliquot of 2 µL from all concentration tested onto Nutrient Agar (NA) plates which were then incubated at 30 °C for 24 h. The MBC endpoint was defined as the lowest concentration of the compounds that kill microorganisms where no visible growth of the microorganism tested was observed on the agar plates.

Table S2 Antimicrobial, cytotoxic and nematicidal activities of compounds **1**–**6**.

Compound	Antimicrobial activity (µg/ml)				Cytotoxicity (IC_{50}, µg/ml)		Nematicidal (µg/ml)
	BS	EC	CT	MP	L929	KB3.1	CE
Pigmentosin A (**1**)	**12.5**	NA	NA	NA	NA	**17**	NA
Pigmentosin B (**2**)	**100**	NA	NA	NA	NA	NA	NA
Glucoasperfuran (**3**)	NA	NA	NA	NA	NA	NA	NA
Beauverolide N (**4**)	NA	NA	NA	NA	NA	**16**	NA
Beauverolide I (**5**)	NA	NA	NA	NA	NA	**20**[a]	NA
Beauverolide J_b (**6**)	NA	NA	NA	NA	NA	NA	NA
Ciprofloxacin	0.52	0.52					
Nystatin			0.52	8.33			
Epothilone A					0.0038		
Epothilone B						0.00022	
Ivermectin							7.5

BS, *Bacillus subtilis* DSM10; EC, *Escherichia coli* DSM498; CT, *Candida tenuis* MUCL 29892; MP, *Mucor plumbeus* MUCL 49355; L929, Murine fibroblast cell line L929; KB3.1, HeLa cell line KB3.1; CE, *Caenorhabditis elegans*, NA, No activity; [a], Proliferation inhibition, no altered or dead cells.

Table S3 Anti-biofilm activity of compounds **1**–**6**, bacteriostatic and bactericidal activities of active compounds.

Concentration (µg/ml)	Anti-biofilm activity (µg/ml)		Bacteriostatic and bactericidal activities against *S. aureus*									
			Bacteriostatic activity									MBC (µg/ml)
			MIC$_{90}$ (µg/ml)	Cell growth (%) at each concentration (µg/ml)								
	SA	PA		250	125	62.5	31.3	15.6	7.8	3.9	1.9	
Pigmentosin A (**1**)	**1.9**	NA	>250	32.5%	23.8%	22.6%	25.4%	30.3%	32.8%	53.2%	89.5%	>250
Pigmentosin B (**2**)	**15.6**	NA	>250	26.8%	29.9%	42.8%	40.9%	54.3%	84.9%	94.4%	100%	>250
Glucoasperfuran (**3**)	NA	NA										
Beauverolide N (**4**)	**250**	NA										
Beauverolide I (**5**)	NA	NA										
Beauverolide J_b (**6**)	NA	NA										
Tetracycline	1.9	1.9										
MeOH	NA	NA	>250	100%	100%	100%	100%	100%	100%	100%	100%	ND
DMSO	NA	NA	>250	100%	98.2%	98.2%	98.7%	98.3%	100%	99.7%	100%	ND

SA, *Staphylococcus aureus* ATCC25923; PA, *Pseudomonas aeruginosa* PA14; NA, No activity; ND, Not done

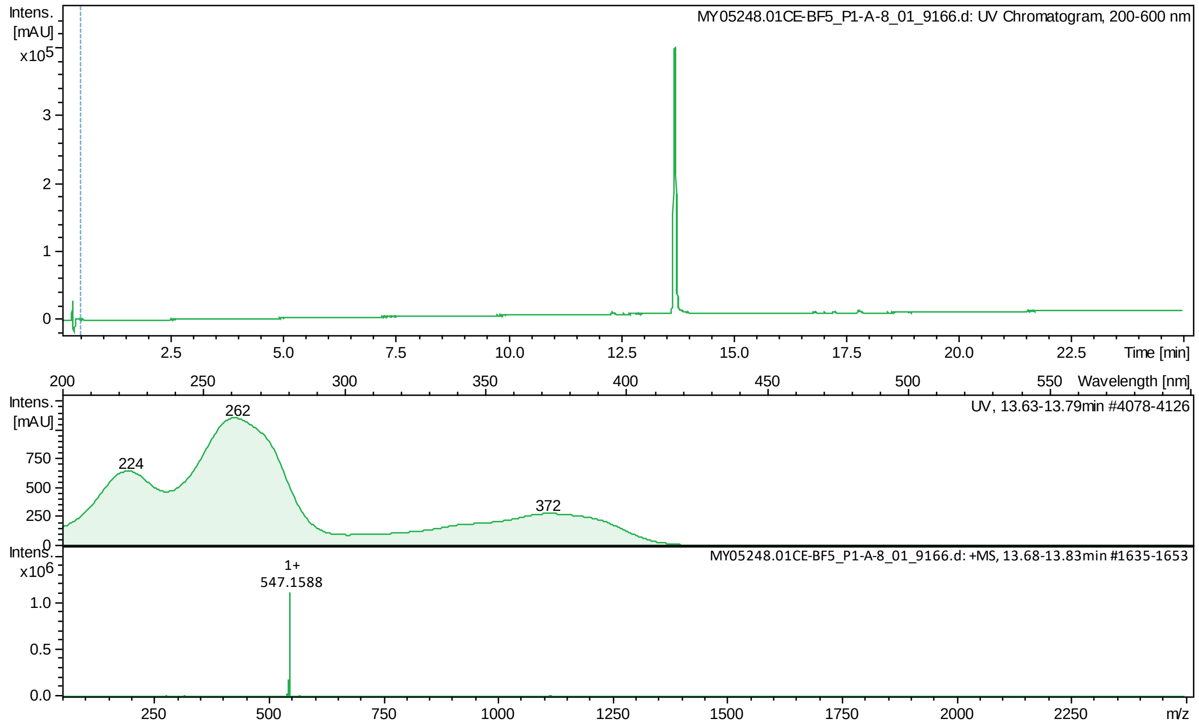

Figure S2. HRMS spectra of pigmentosin A (**1**).

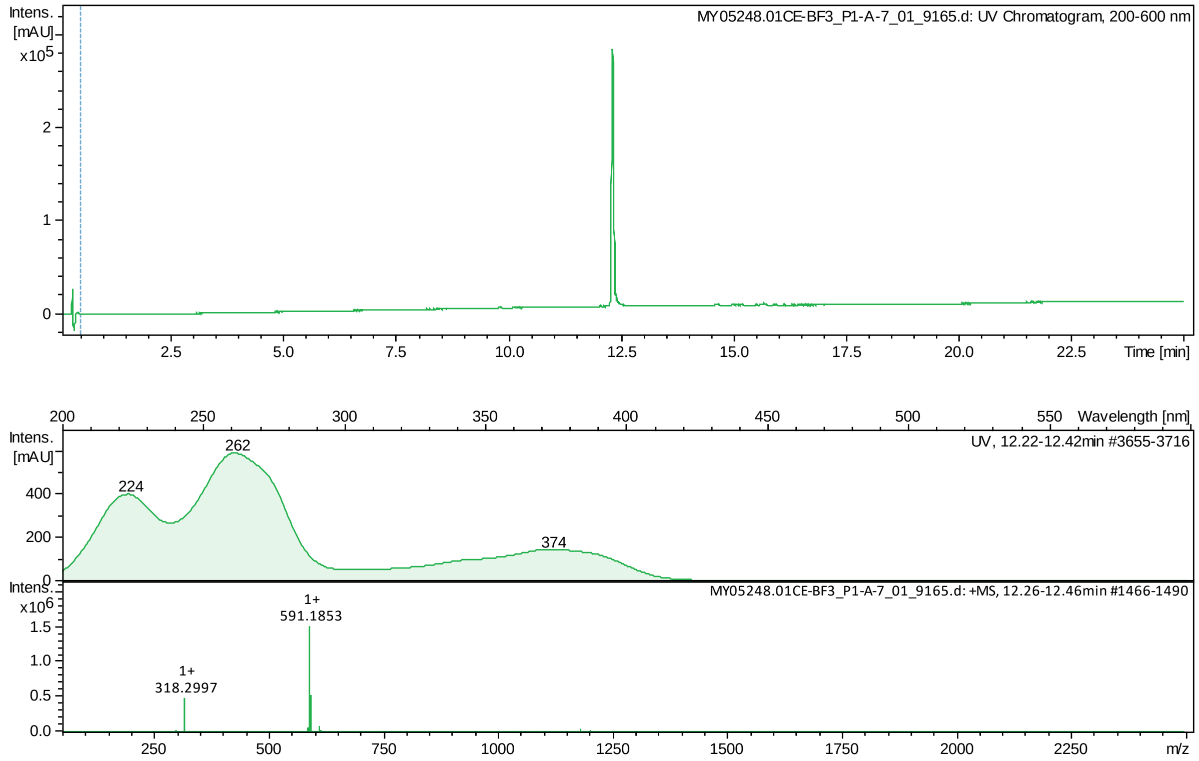

Figure S3. HRMS spectra of pigmentosin B (**2**).

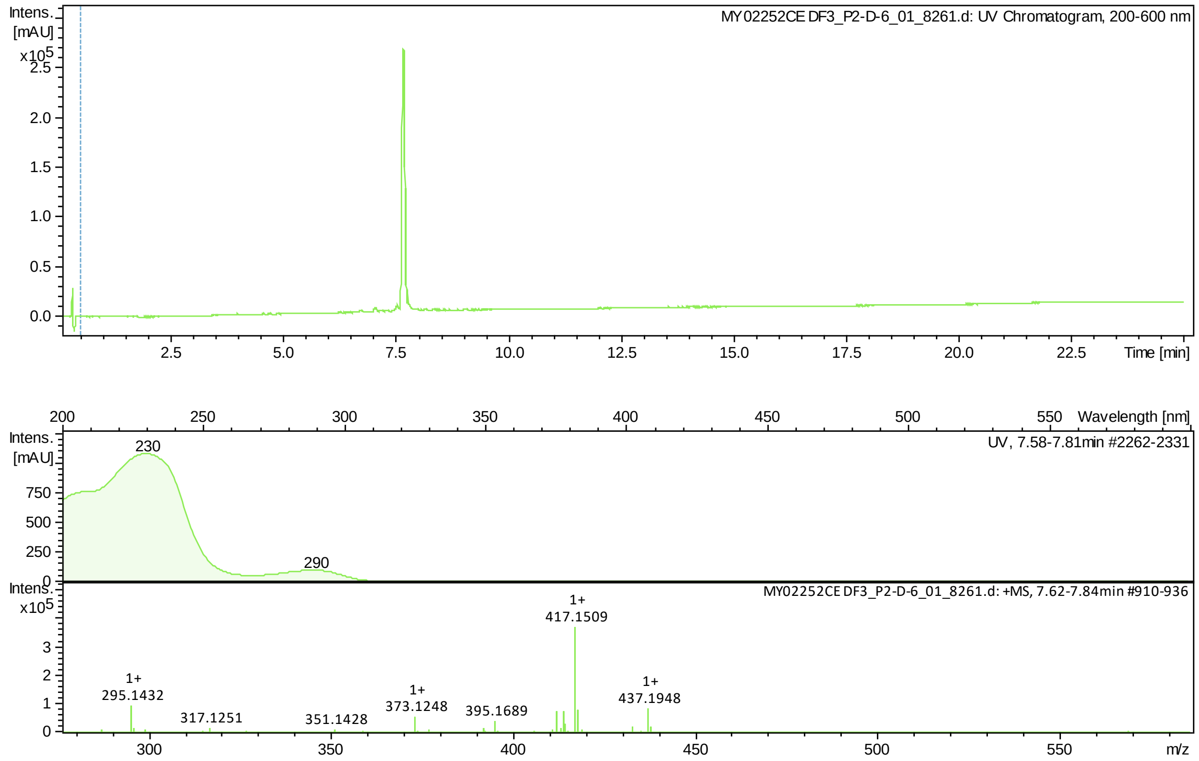

Figure S4. HRMS spectra of glycoasperfuran (**3**).

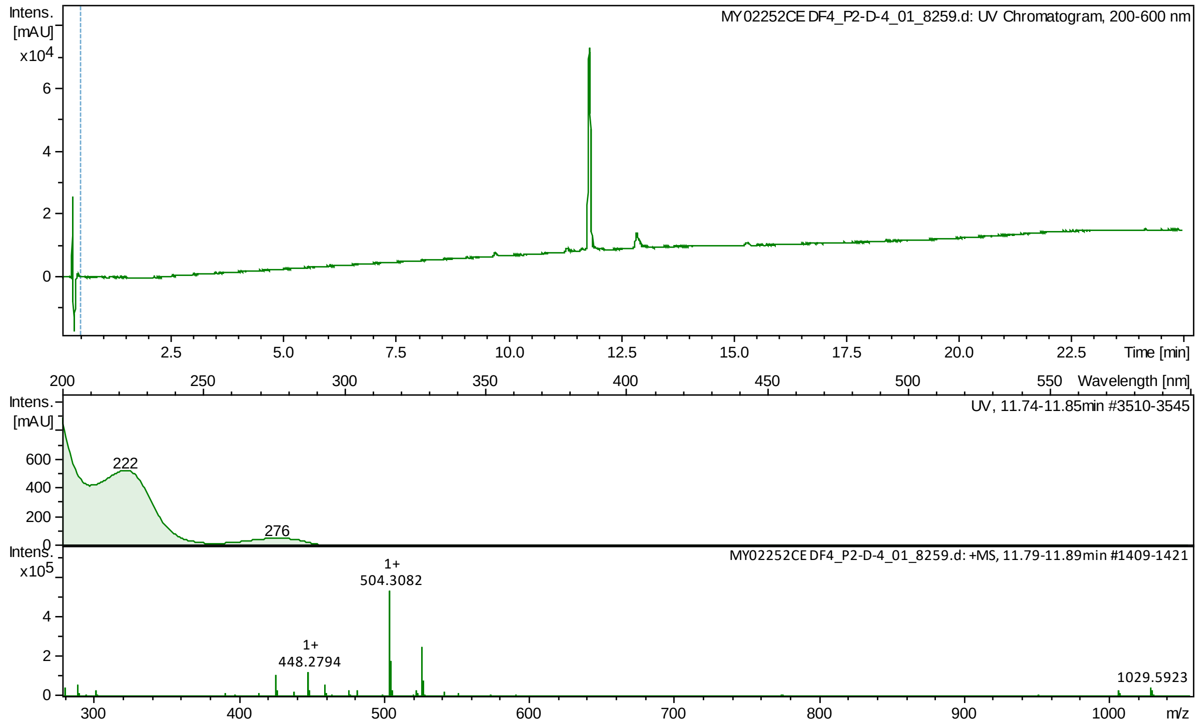

Figure S5. HRMS spectra of beauverolide N (**4**).

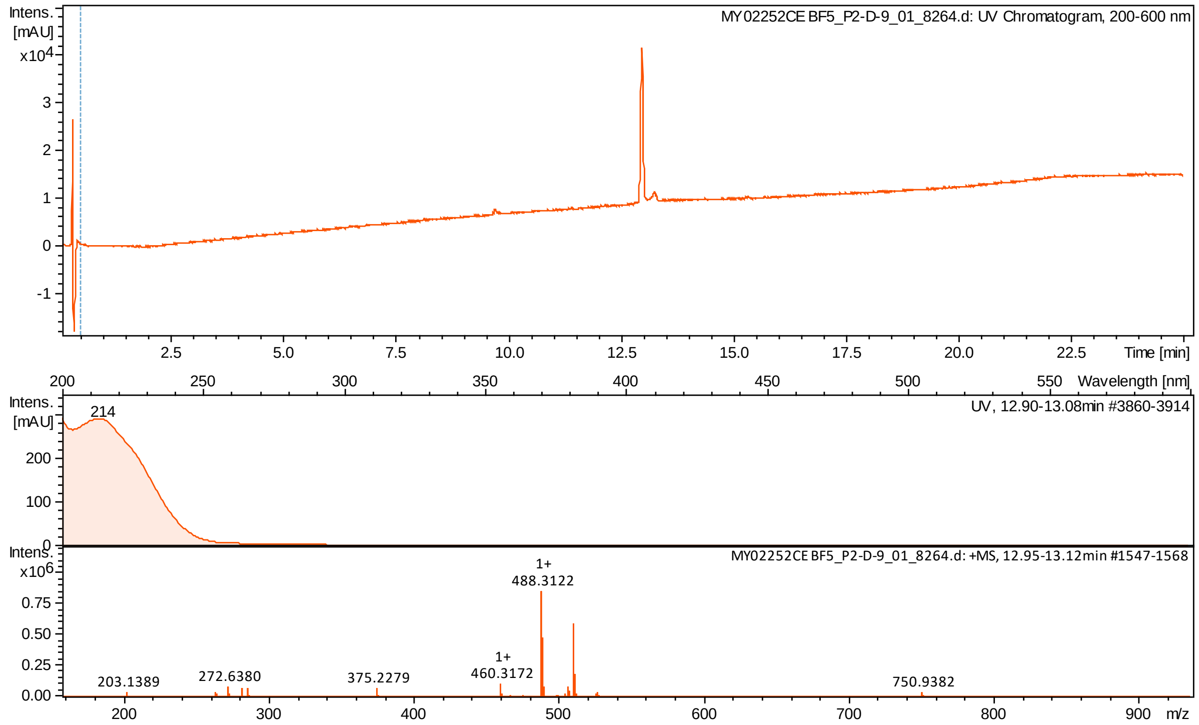

Figure S6. HRMS spectra of beauverolide I (**5**).

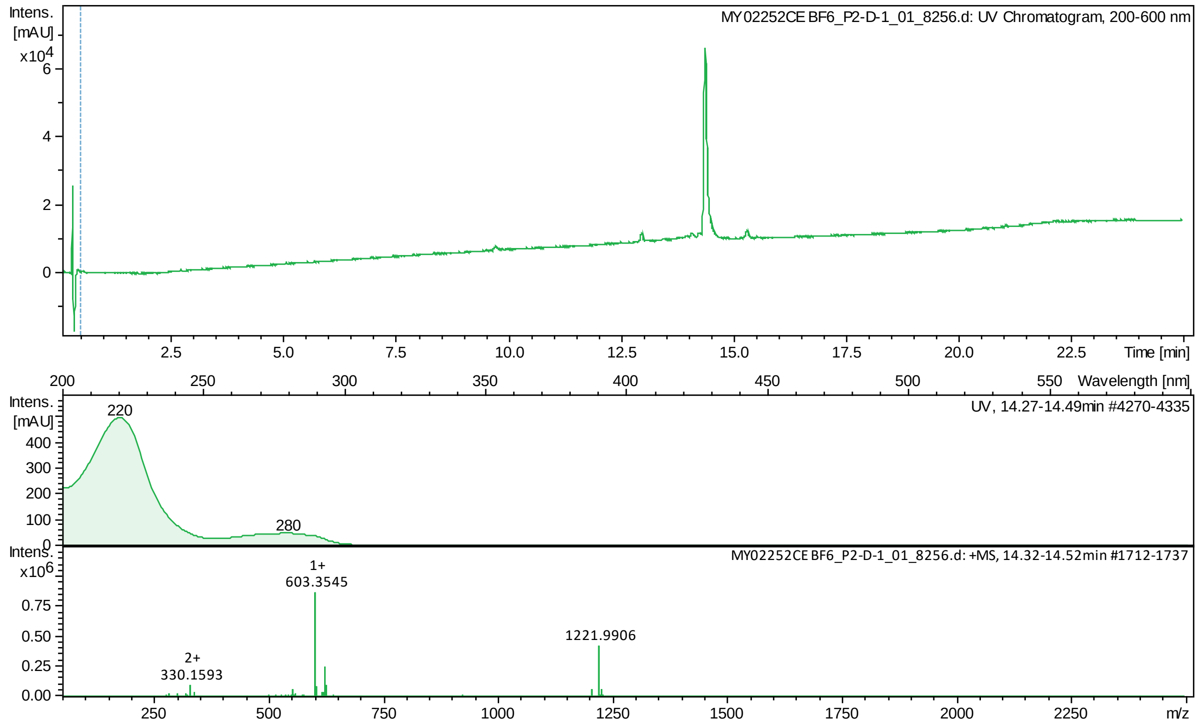

Figure S7. HRMS spectra of beauverolide J_b (**6**).

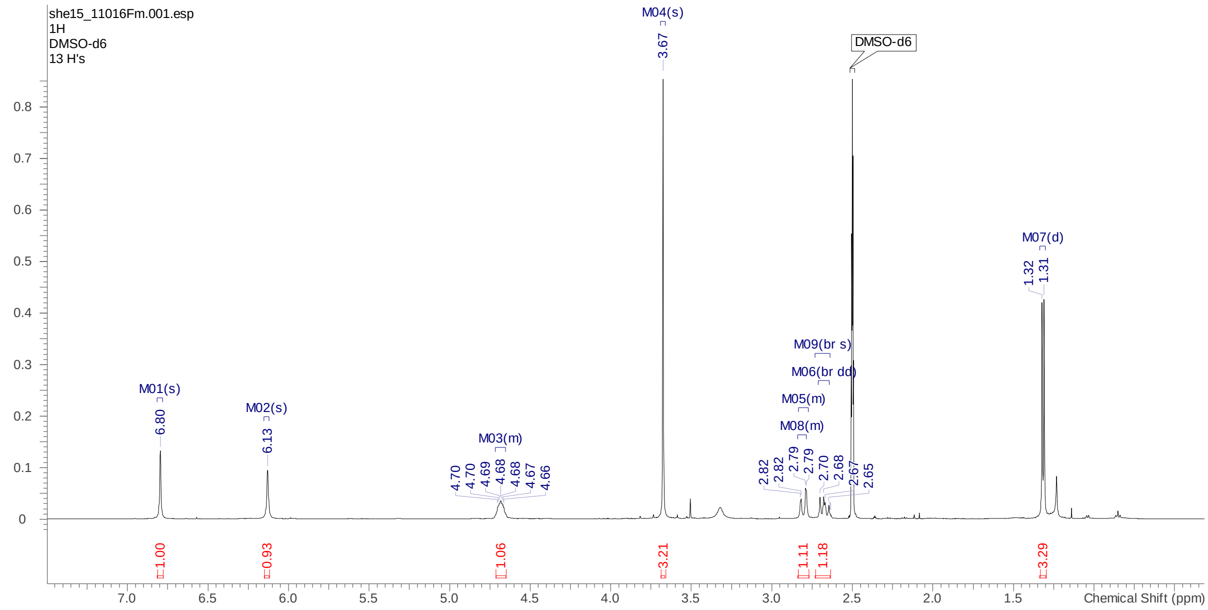

Figure S8.^{1}H NMR spectrum for pigmentosin A (**1**) (500 MHz, DMSO-d_6).

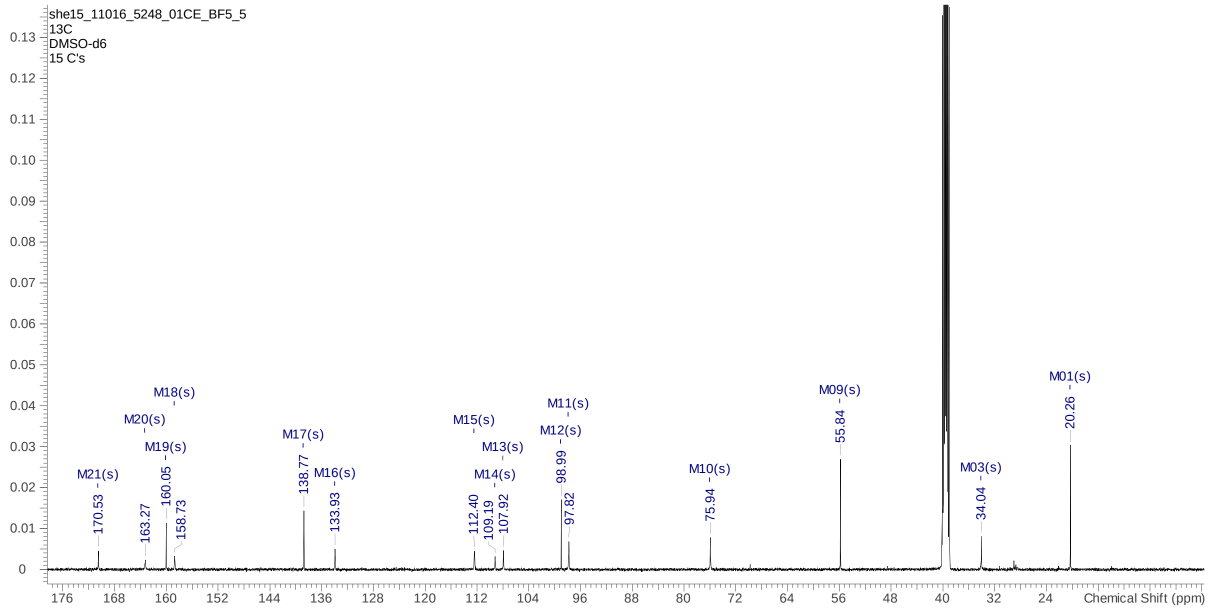

Figure S9.^{13}C NMR spectrum for pigmentosin A (**1**) (125 MHz, DMSO-d_6).

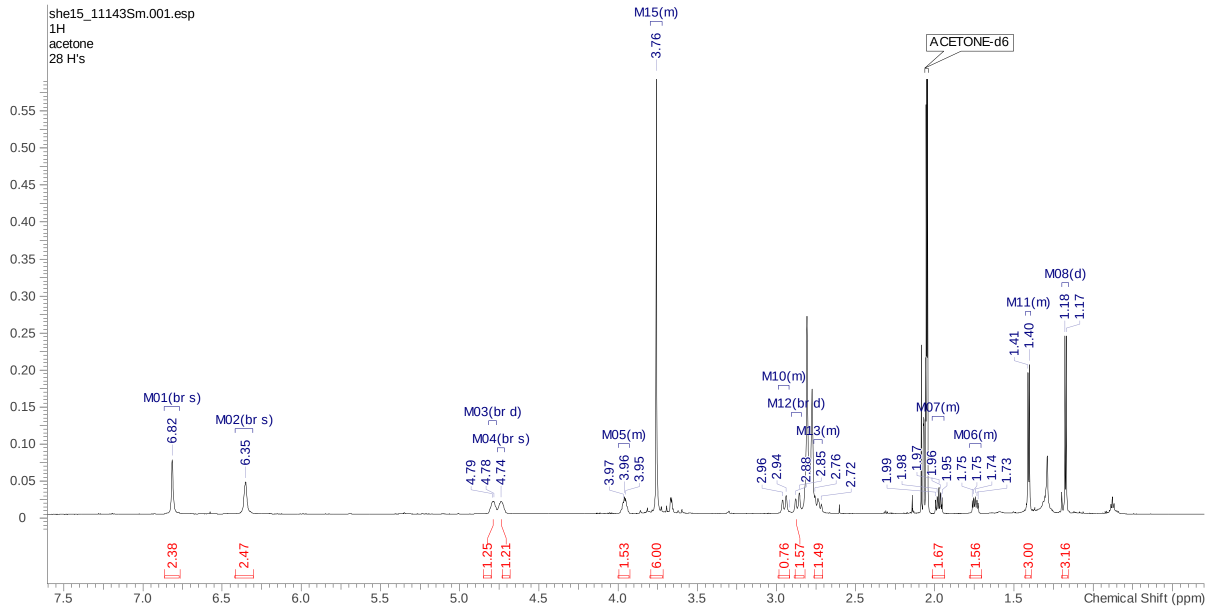

Figure S10.^{1}H NMR spectrum for pigmentosin B (**2**) (500 MHz, acetone-d_6).

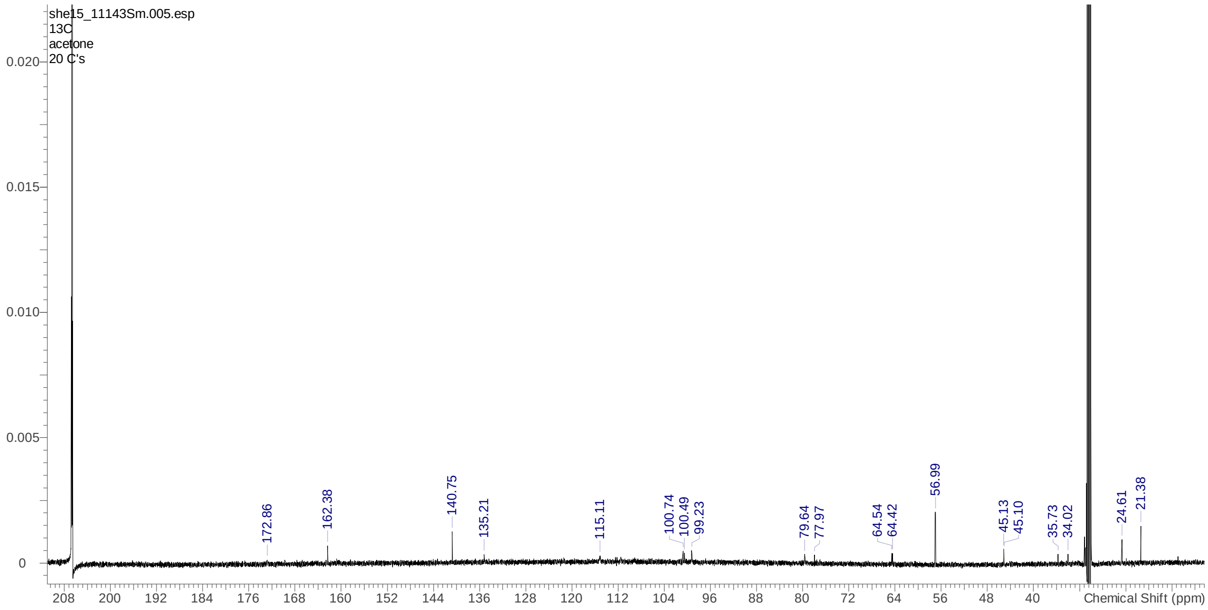

Figure S11.^{13}C NMR spectrum for pigmentosin B (**2**) (125 MHz, acetone-d_6).

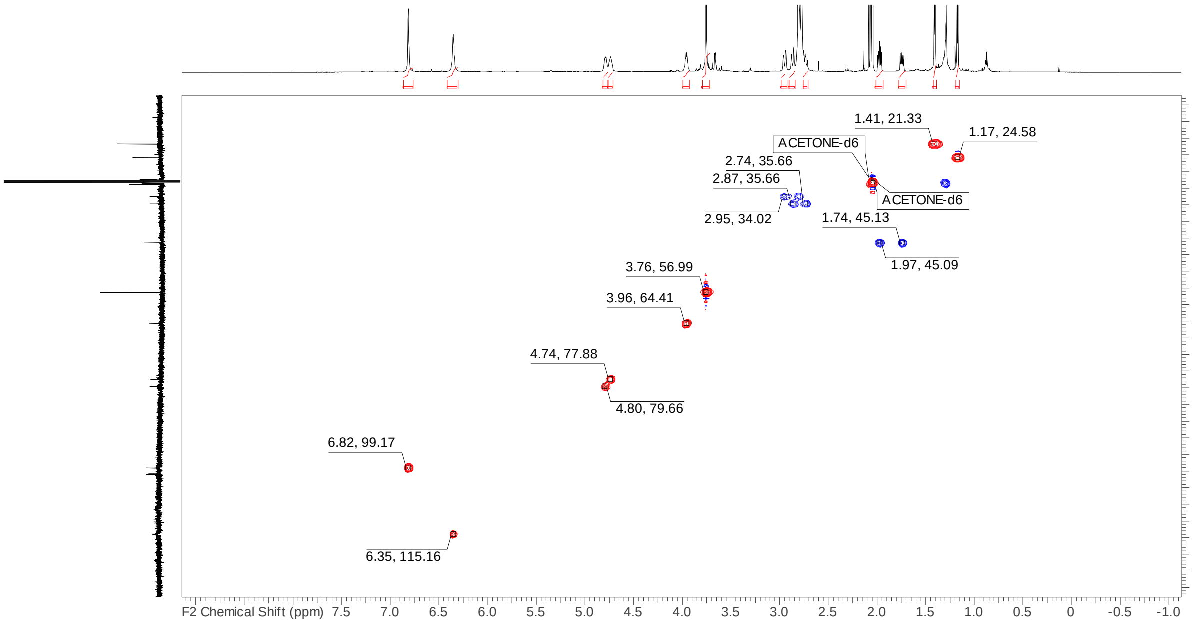

Figure S12.HSQC NMR spectrum for pigmentosin B (**2**) (500 MHz, acetone-d_6).

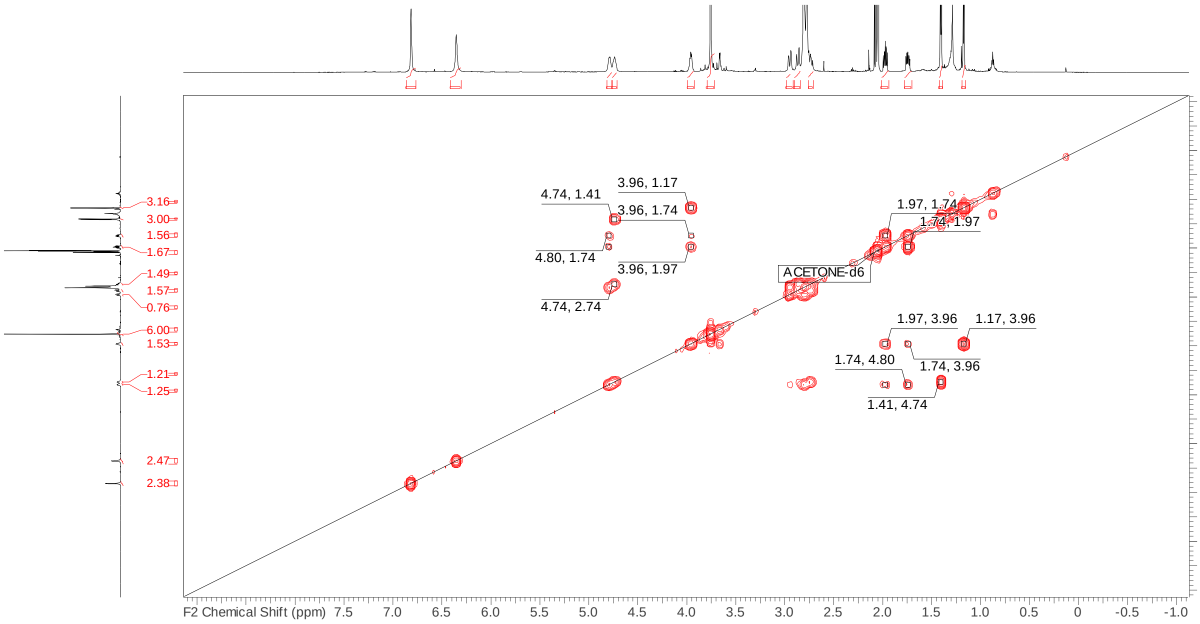

Figure S13.COSY NMR spectrum for pigmentosin B (**2**) (500 MHz, acetone-d_6).

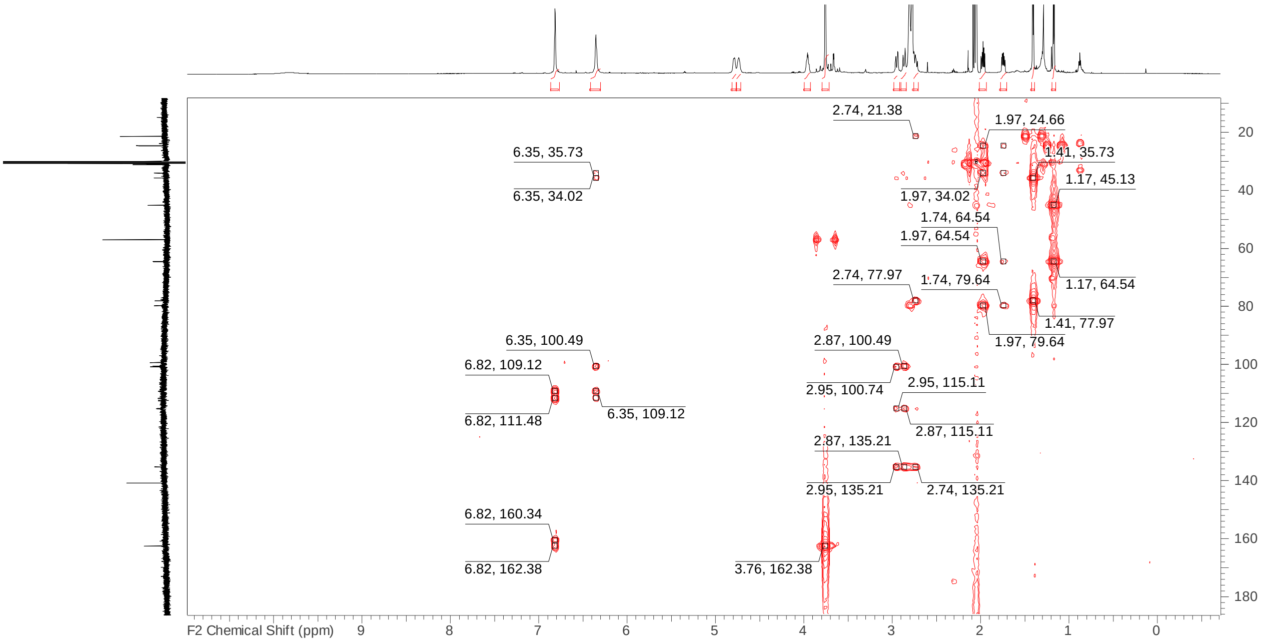

Figure S14.HMBC NMR spectrum for pigmentosin B (**2**) (500 MHz, acetone-*d*6).

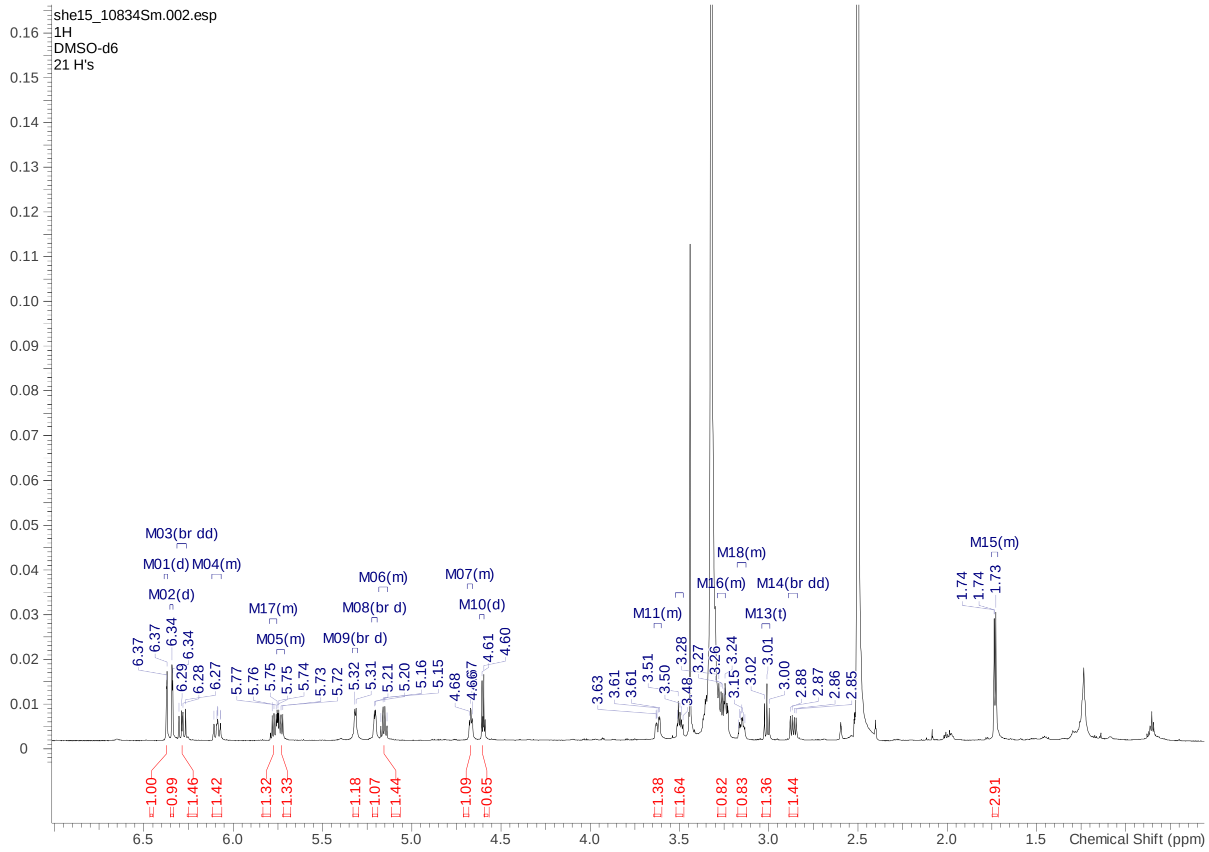

Figure S15.^{1}H NMR spectrum for glucoasperfuran (**3**) (700 MHz, DMSO-*d*6).

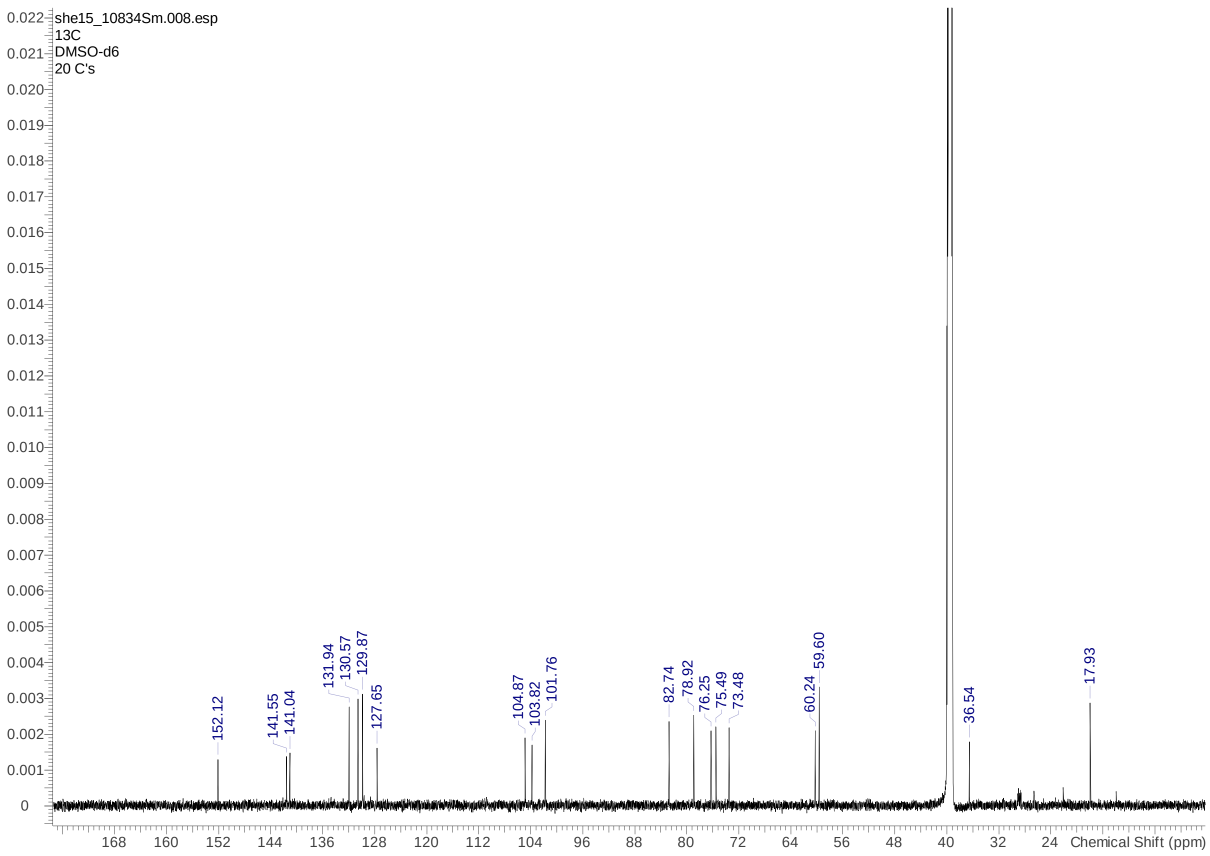

Figure S16.^{13}C NMR spectrum for glucoasperfuran (**3**) (175 MHz, DMSO-d_6).

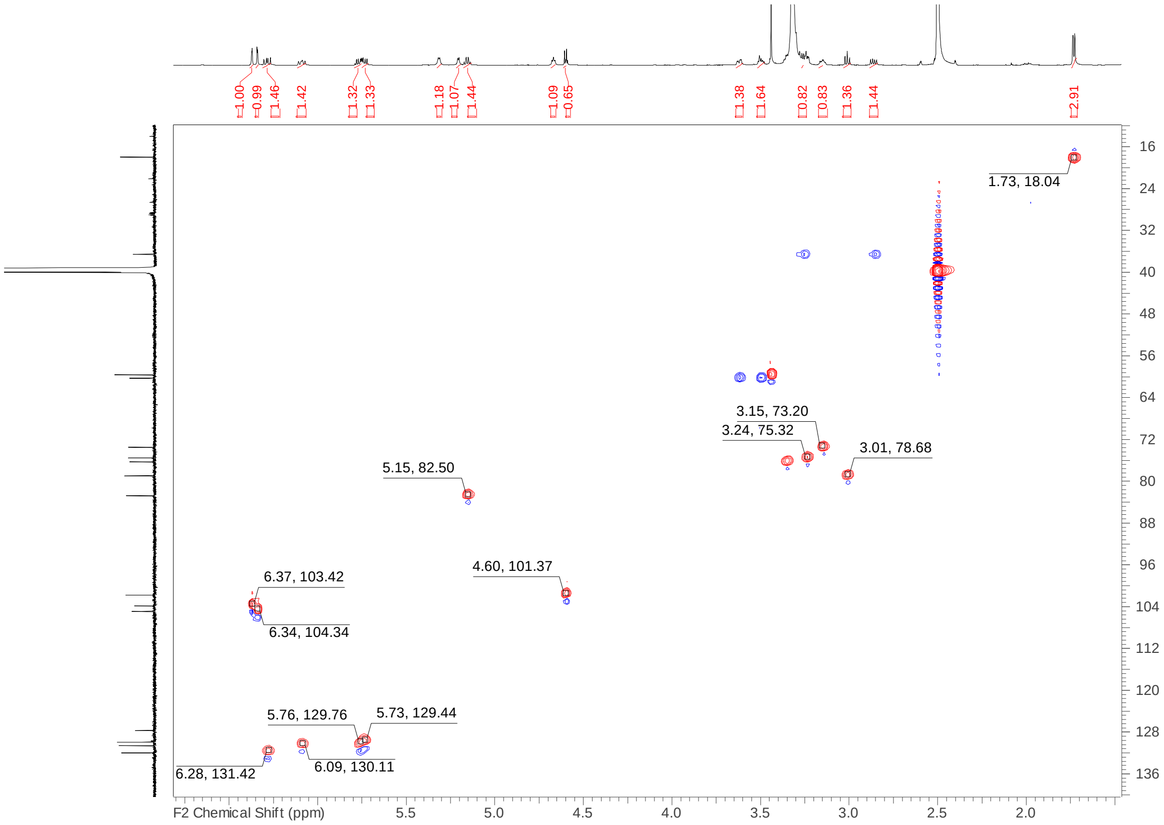

Figure S17. HSQC NMR spectrum for glucoasperfuran (**3**) (700 MHz, DMSO-d_6).

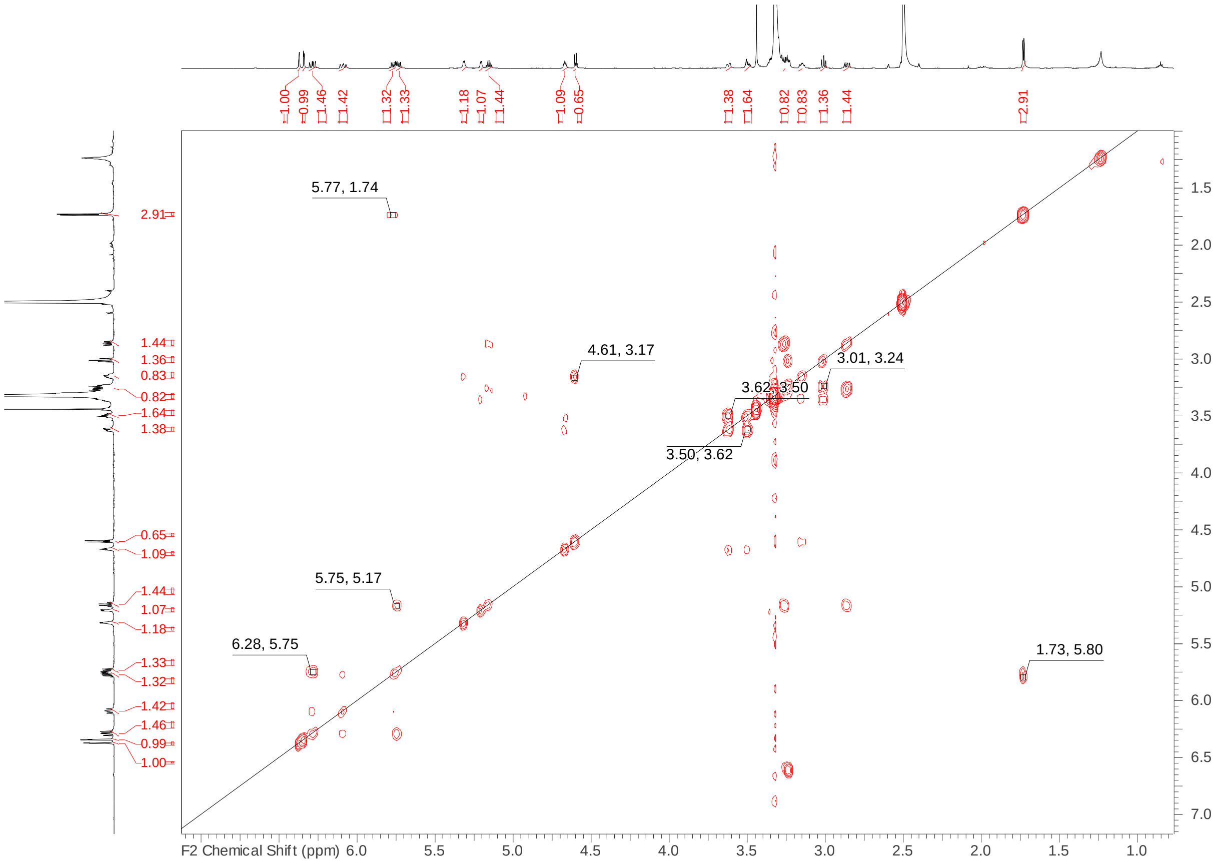

Figure S18.COSY NMR spectrum for glucoasperfuran (**3**) (700 MHz, DMSO-*d*6).

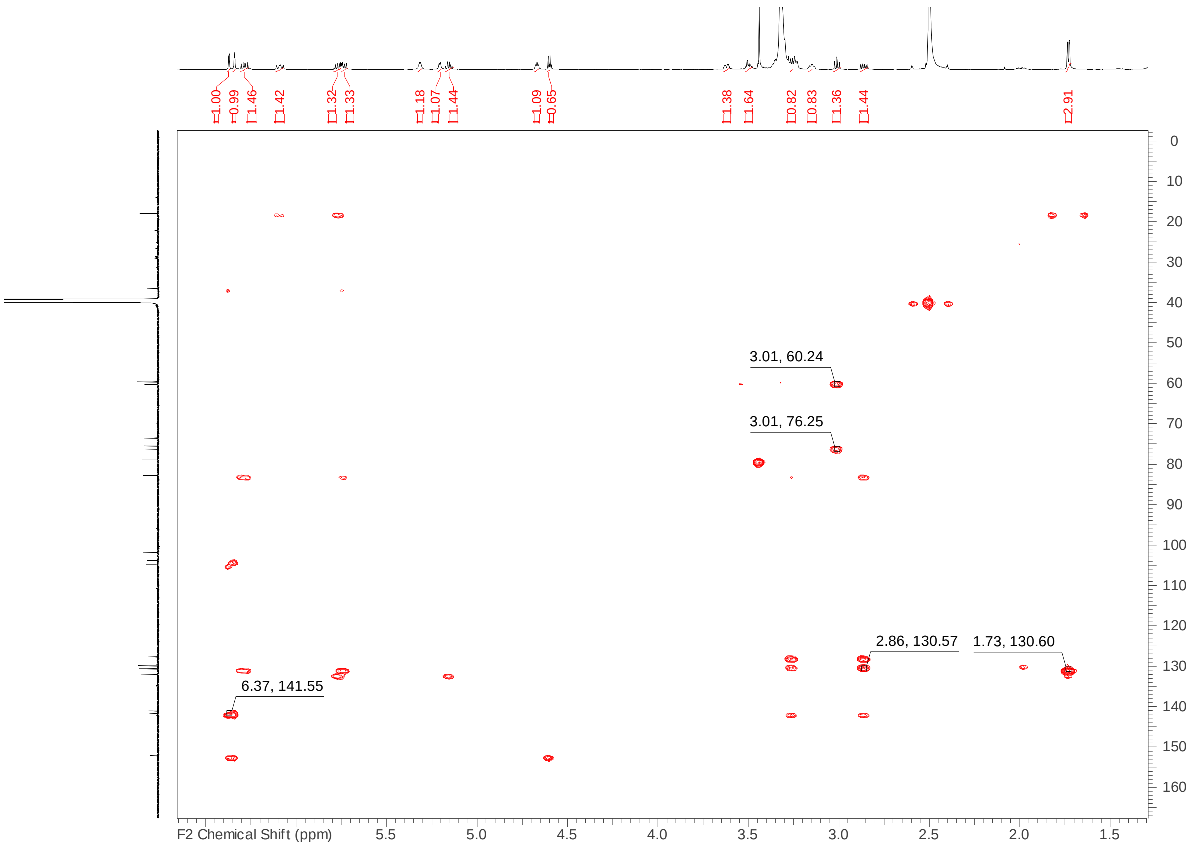

Figure S19.HMBC NMR spectrum for glucoasperfuran (**3**) (700 MHz, DMSO-*d*6).

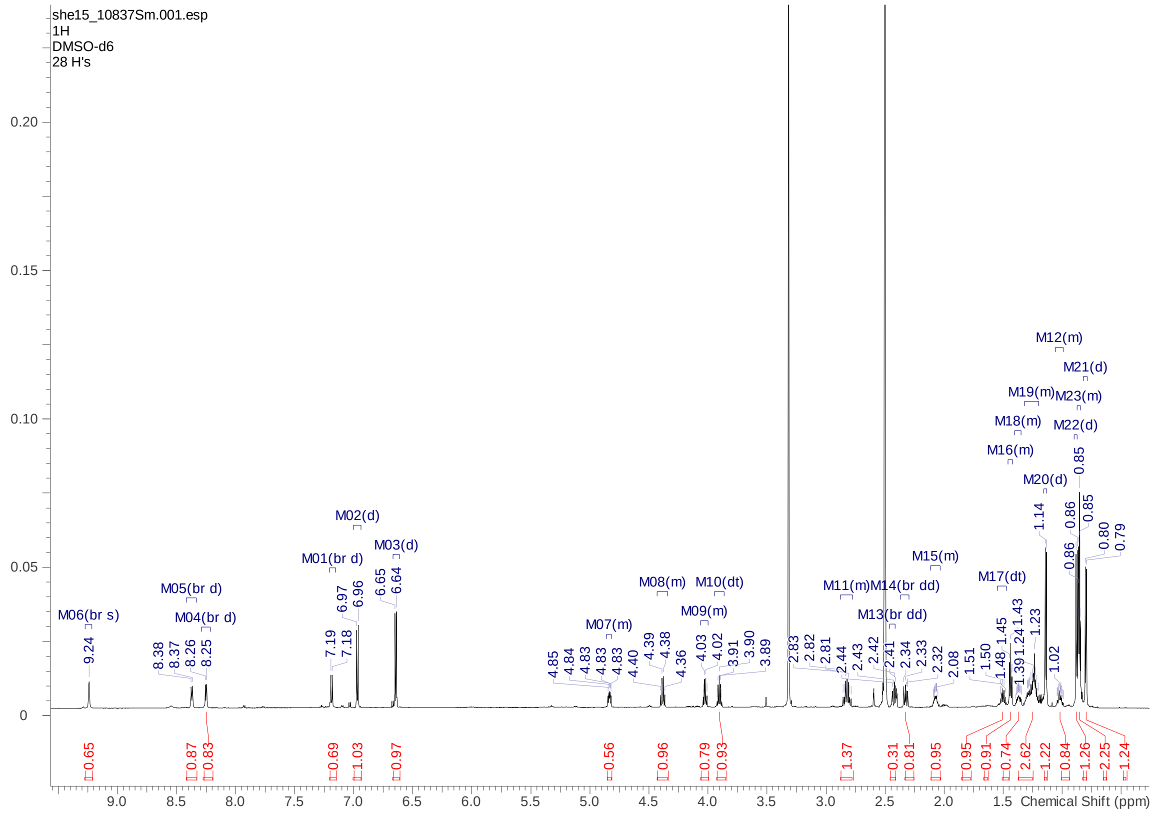

Figure S20.^{1}H NMR spectrum forbeauverolide N (**4**) (700 MHz, DMSO-*d*6).

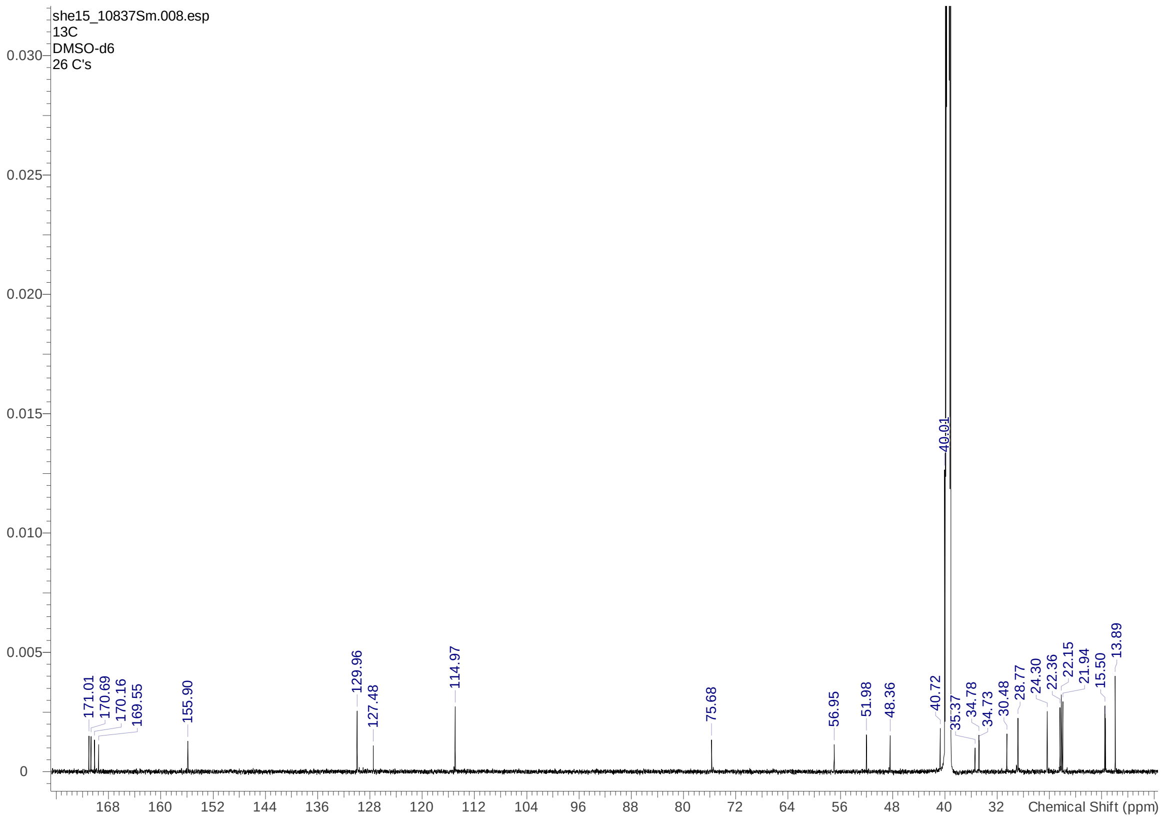

Figure S21.^{13}C NMR spectrum forbeauverolide N (**4**) (175 MHz, DMSO-*d*6).

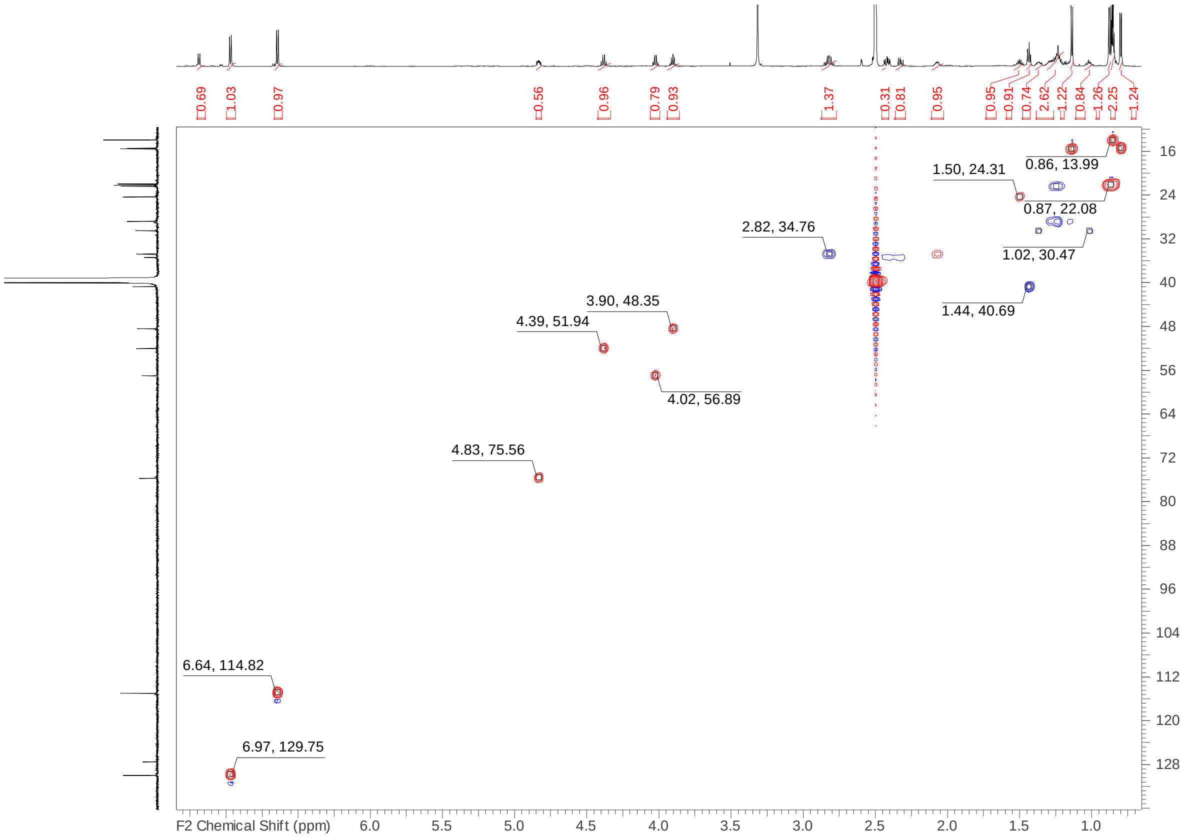

Figure S22. HSQC NMR spectrum forbeauverolide N (**4**) (700 MHz, DMSO-d_6).

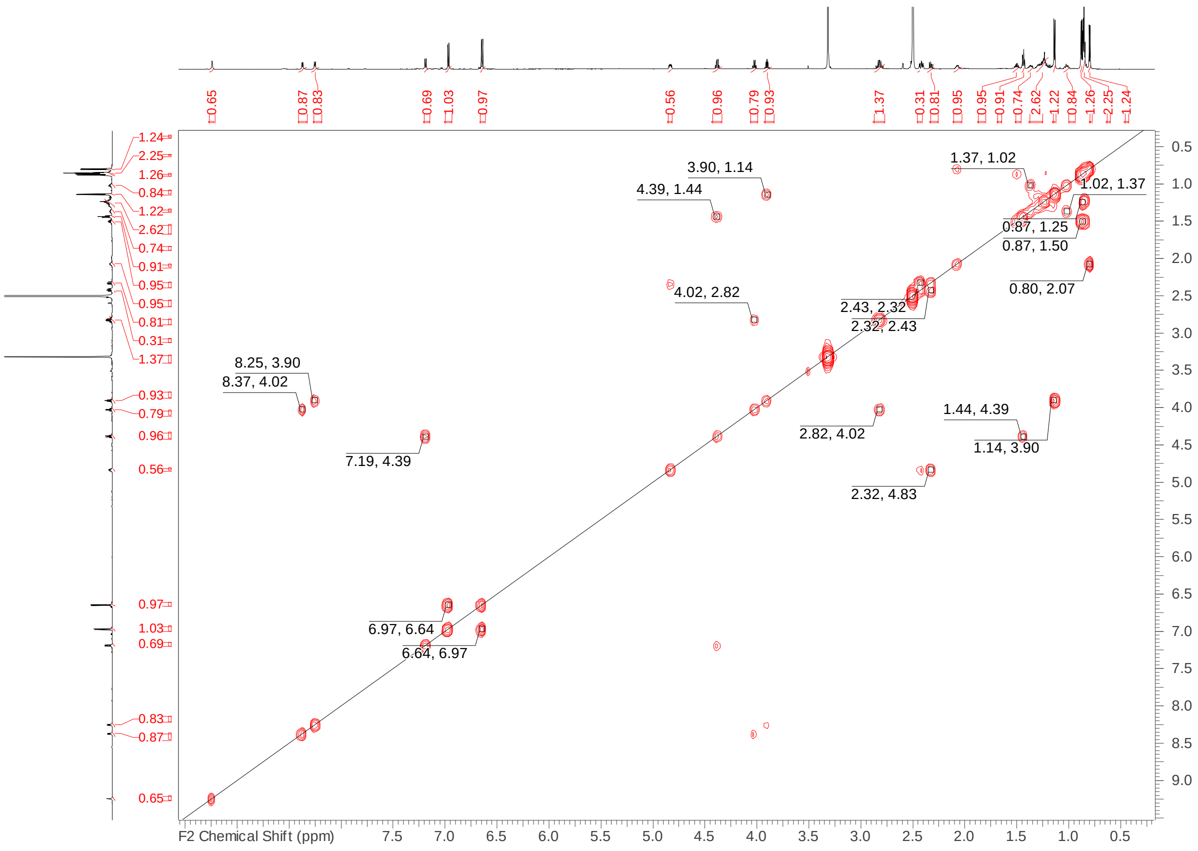

Figure S23.COSY NMR spectrum forbeauverolide N (**4**) (700 MHz, DMSO-d_6).

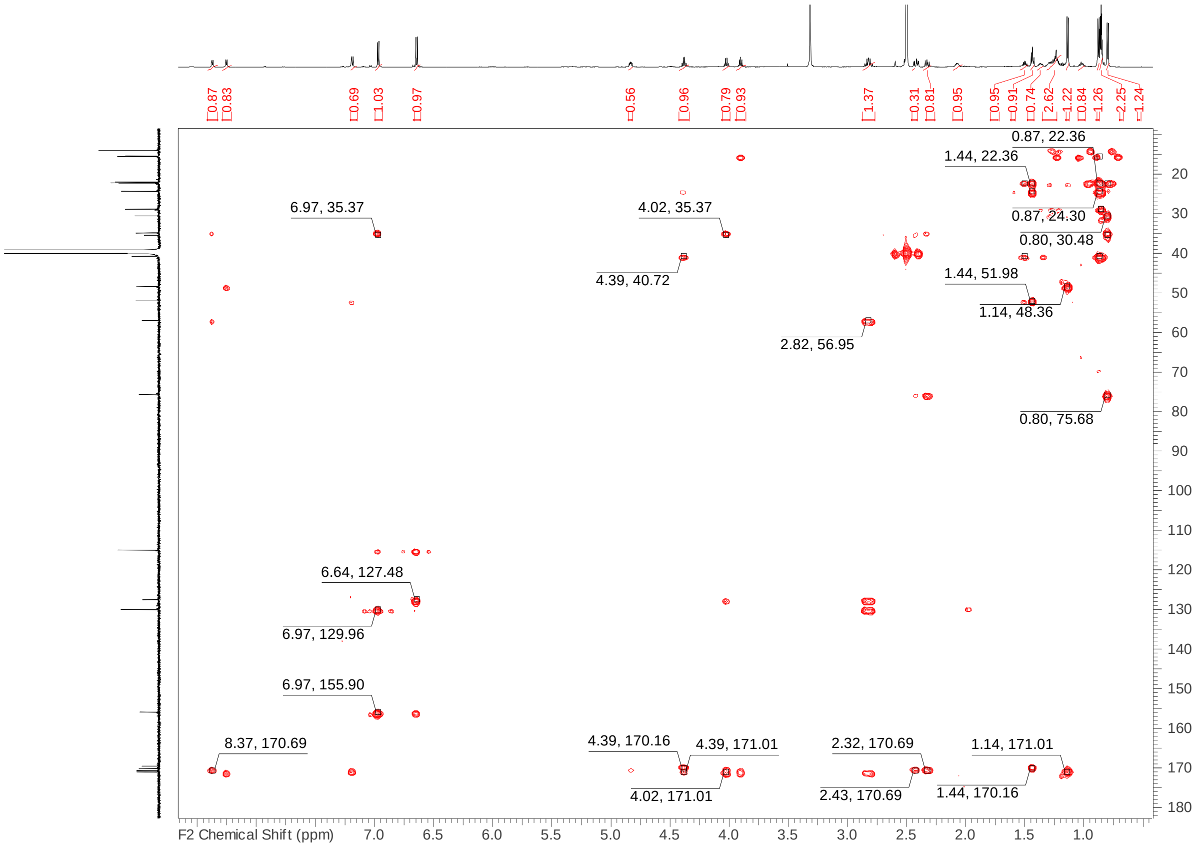

Figure S24. HMBC NMR spectrum forbeauverolide N (**4**) (700 MHz, DMSO-d_6).

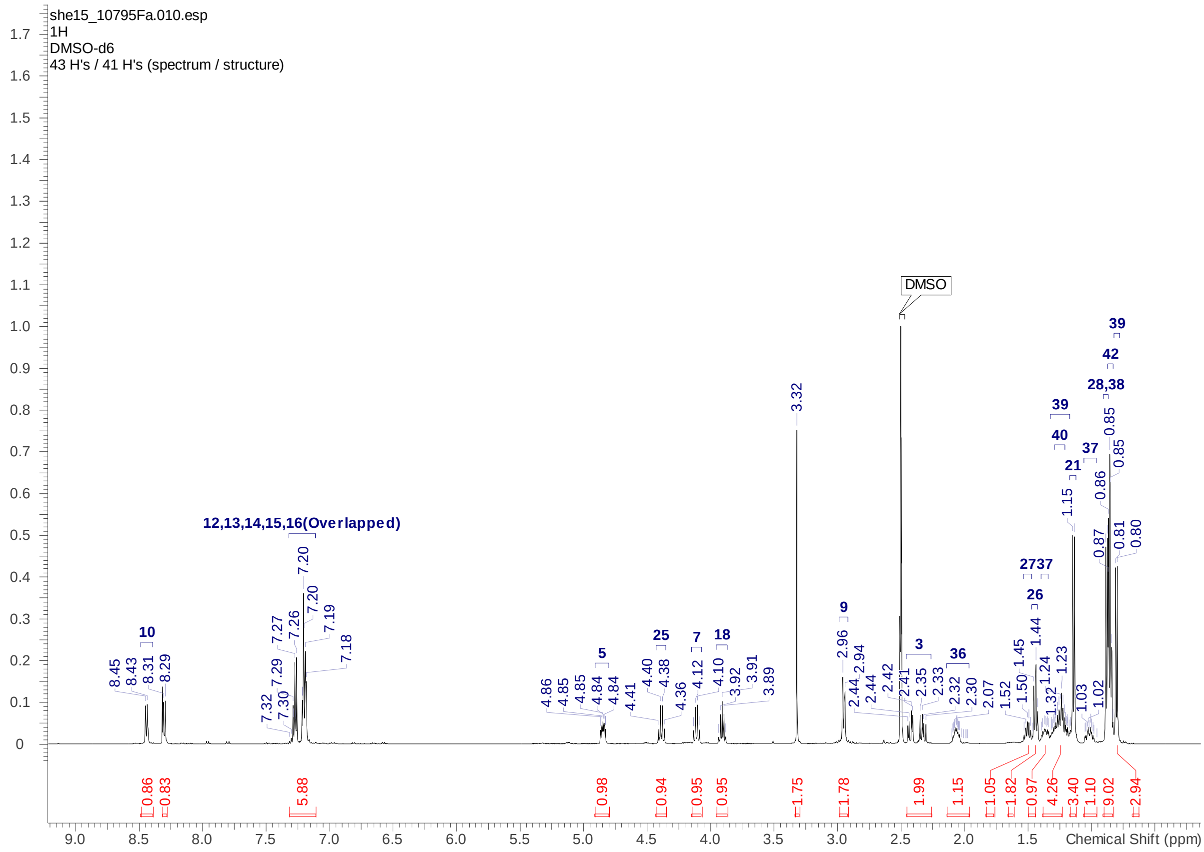

Figure S25.^{1}H NMR spectrum forbeauverolide I (**5**) (500 MHz, DMSO-d_6).

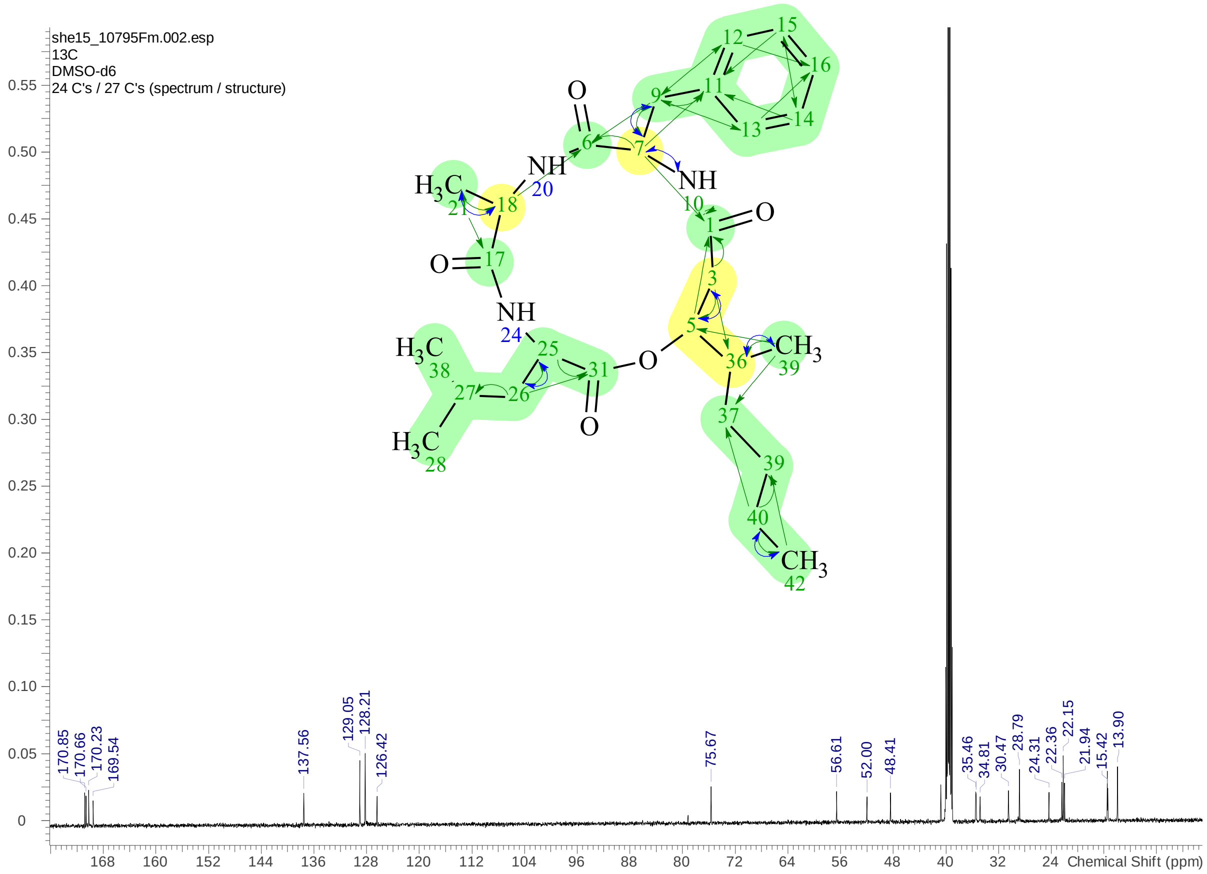

Figure S26. ^{13}C NMR spectrum forbeauverolide I (**5**) (125 MHz, DMSO-d_6).

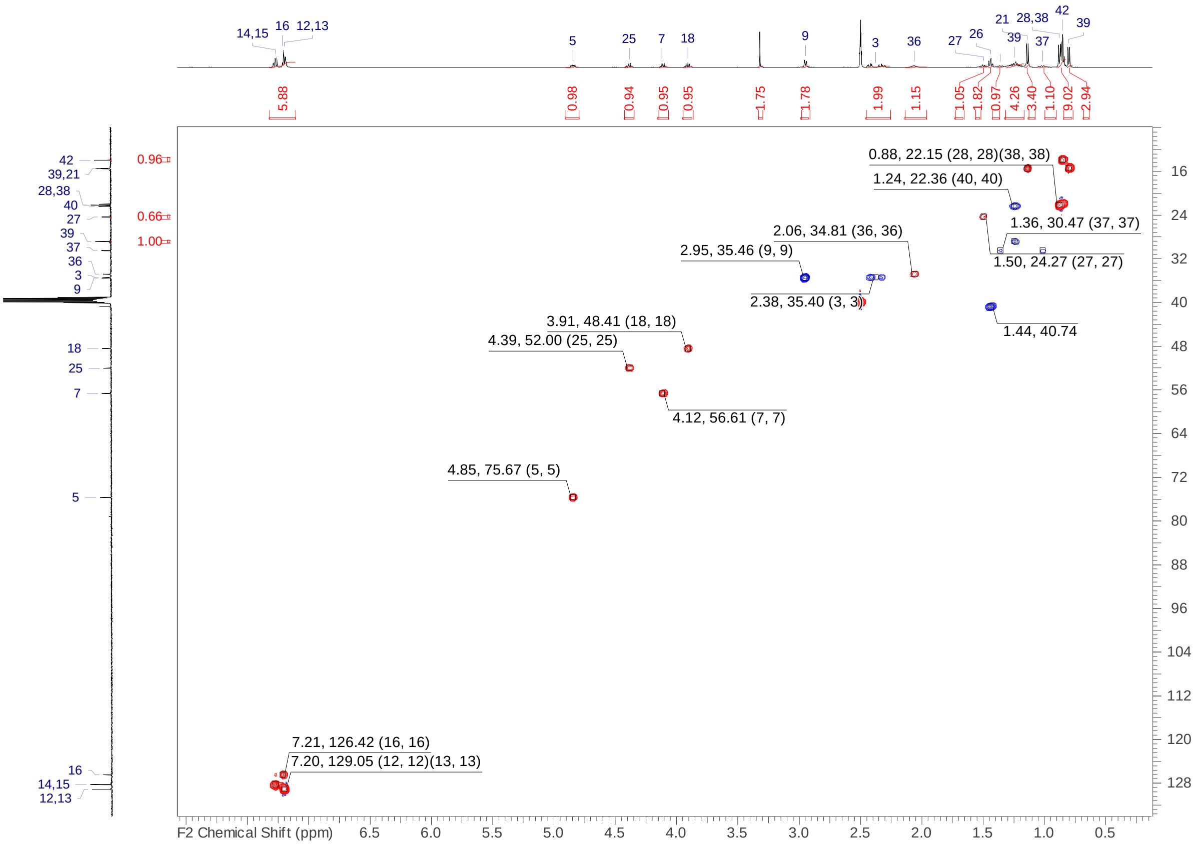

Figure S27. HSQC NMR spectrum forbeauverolide I (**5**) (500 MHz, DMSO-d_6).

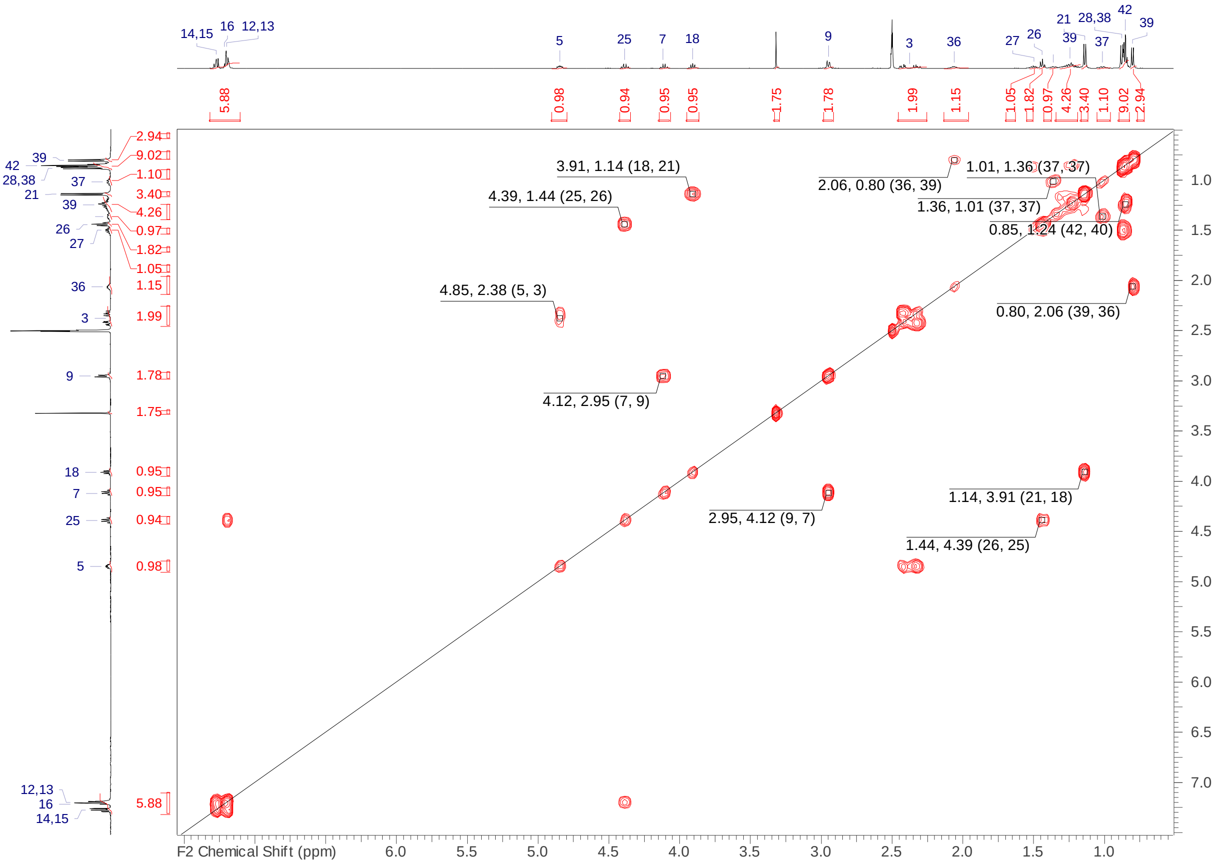

Figure S28.COSY NMR spectrum forbeauverolide I (**5**) (500 MHz, DMSO-d_6).

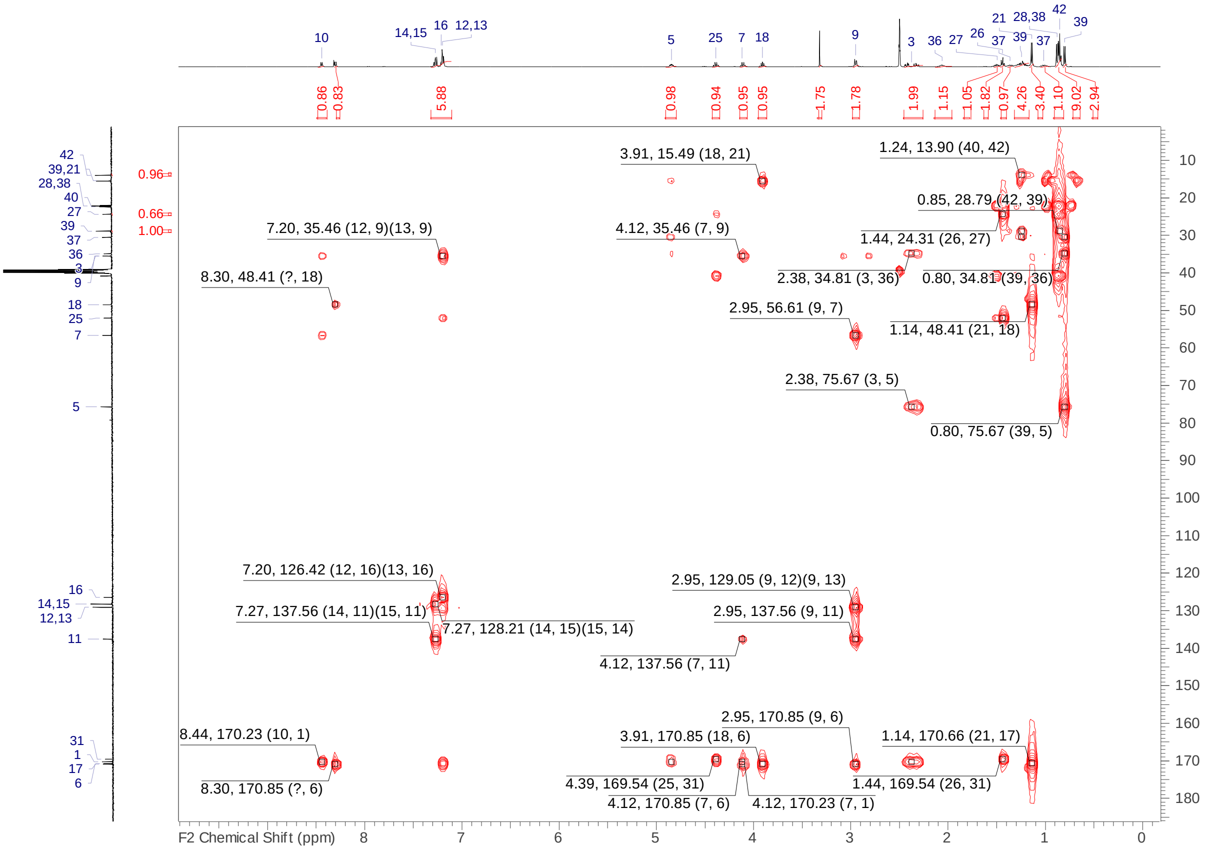

Figure S29. HMBC NMR spectrum forbeauverolide I (**5**) (500 MHz, DMSO-d_6).

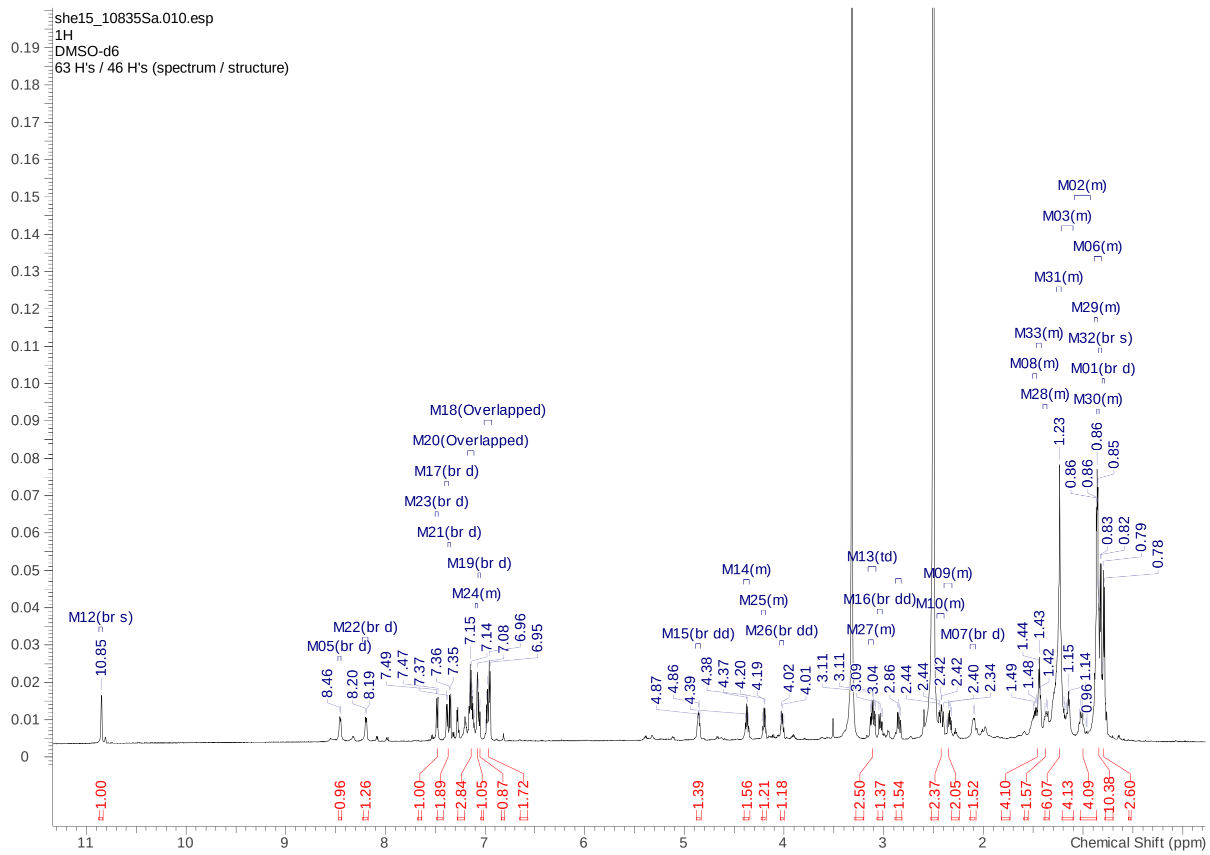

Figure S30.^{1}H NMR spectrum forbeauverolide J_b (**6**) (700 MHz, DMSO-d_6).

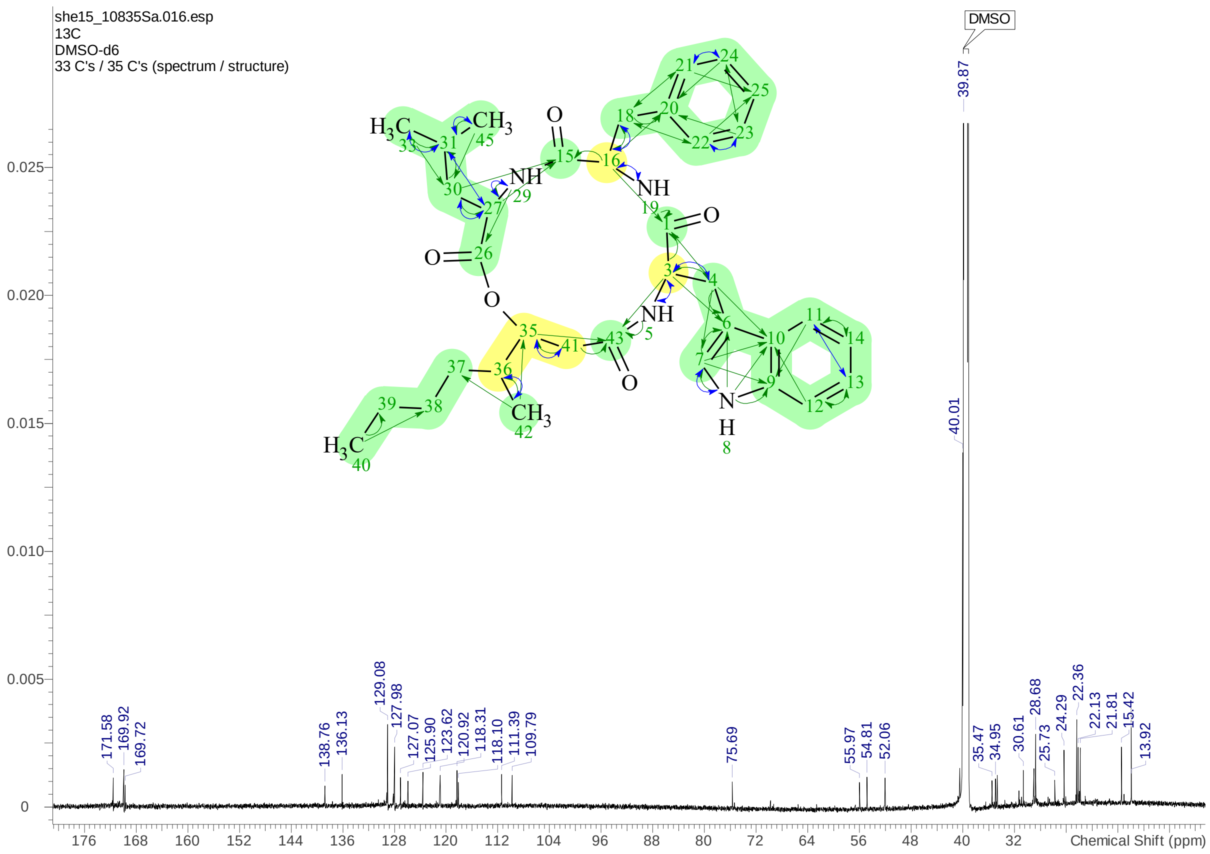

Figure S31.^{13}C NMR spectrum forbeauverolide J_b (**6**) (700 MHz, DMSO-d_6).

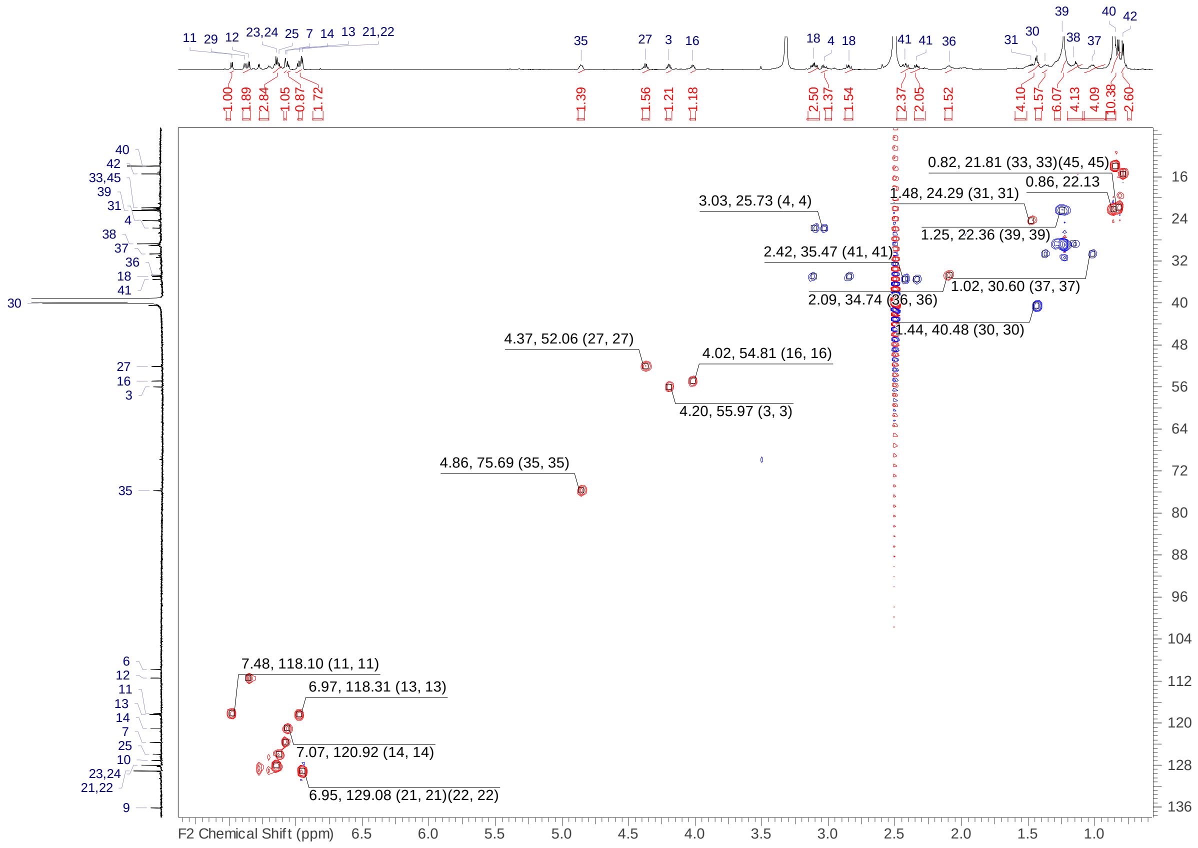

Figure S32. HSQC NMR spectrum forbeauverolide J_b (**6**) (700 MHz, DMSO-d_6).

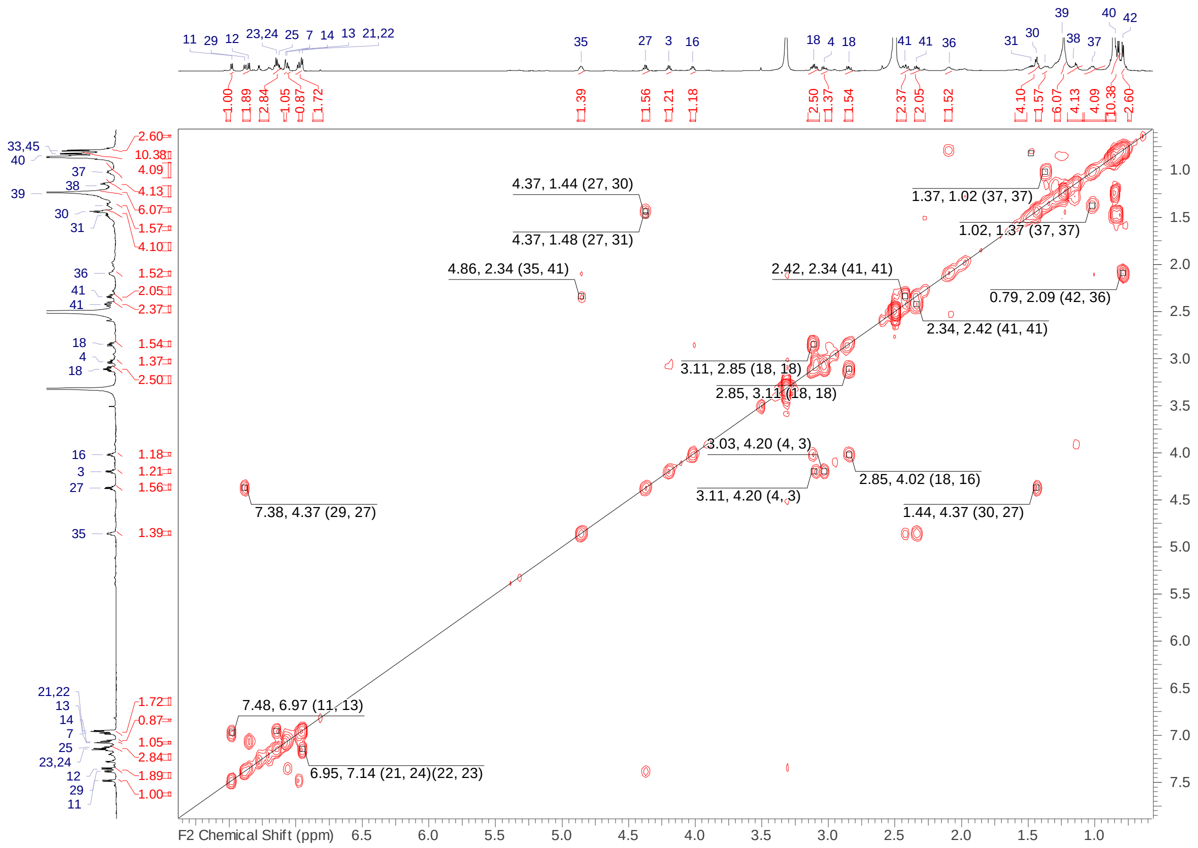

Figure S33.COSY NMR spectrum forbeauverolide J_b (**6**) (700 MHz, DMSO-d_6).

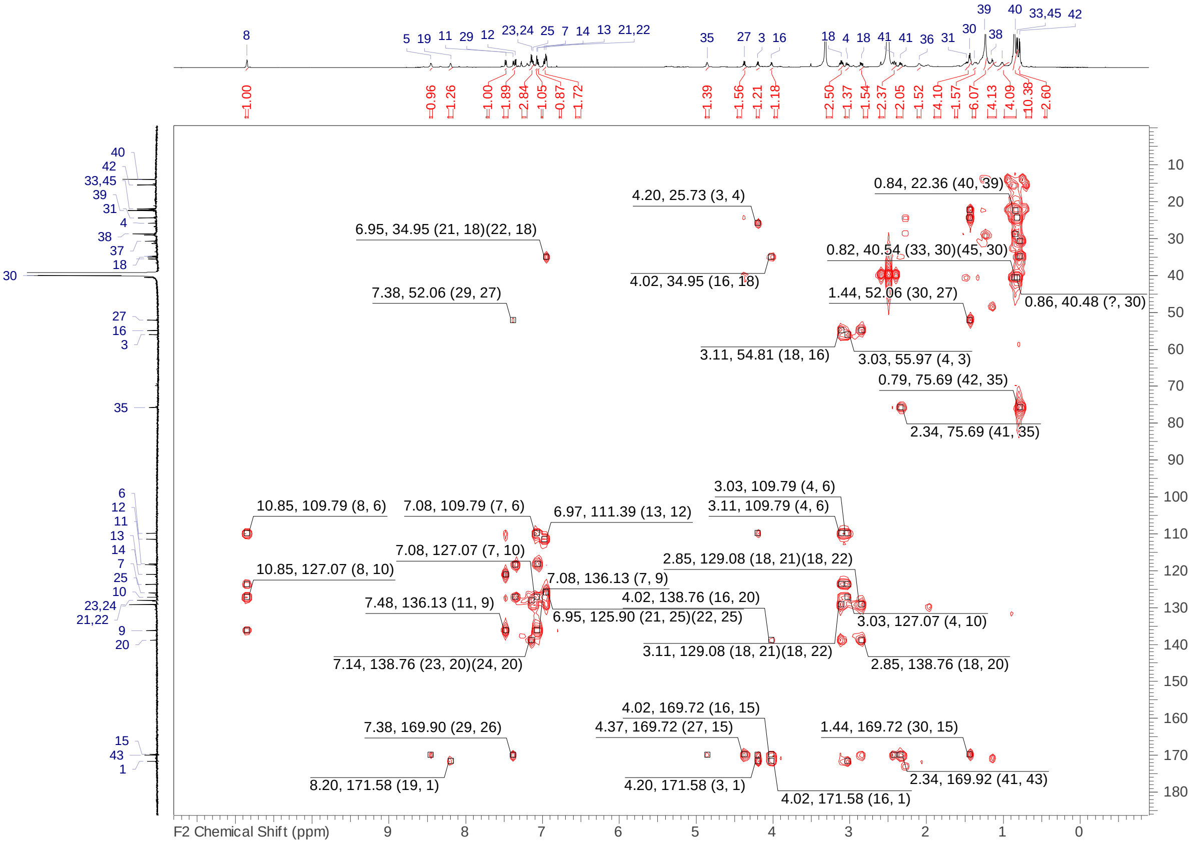

Figure S34. HMBC NMR spectrum forbeauverolide J_b (**6**) (700 MHz, DMSO-d_6).

References

1 W. Kuephadungphan, A.P.G. Macabeo, J.J. Luangsa-ard, K. Tasanathai, D. Thanakitpipattana, S. Phongpaichit, K. Yuyama and M. Stadler, *Mycol. Prog*., 2018, **18**, DOI: 10.1007/s11557-018-1431-4.

2 A. Stamatakis, *Bioinformatics*, 2006, **22**, 2688–2690.

3 M. Kearse, R. Moir, A. Wilson, S. Stones-Havas, M. Cheung, S. Sturrock, S. Buxton, A. Cooper, S. Markowitz, C. Duran and T. Thierer, *Bioinformatics*, 2012, **28**, 1647–1649.

4 W. Kuephadungphan, S.E. Helaly, C. Daengrot, S. Phongpaichit, J.J. Luangsa-ard, V. Rukachaisirikul and M. Stadler, *Molecules*, 2017, **22**, DOI: 10.3390/molecules22071202.

5 M. Stadler, H. Anke, W.-R. Arendholz, F. Hansske, U. Anders, O. Sterner and K.-E. Bergquist, *J. Antibiot*., 1993, **46**, 961–967.

6 S.E. Helaly, W. Kuephadungphan, S. Phongpaichit, J.J. Luangsa-ard, V. Rukachaisirikul and M.Stadler, *Molecules*, 2017, **22**, DOI: 10.3390/molecules22060991.

7 C. Chepkirui, J.C. Matasyoh, C. Decock and M. Stadler, *Phytochem. Lett*., 2017, **20**, 106–110.

8 L.G. Rahme, E.J. Stevens, S.F. Wolfort, J. Shao, R.G. Tompkins and F.M. Ausubel, *Science*, 1995, **268**, 1899–1902.

9 G.A. O'Toole, *J. Vis. Exp*., 2011, **47**, 2437.

10 C. Phukhamsakda, A.P.G. Macabeo, K. Yuyama, K.D. Hyde and M. Stadler, *Molecules*, 2018,**23**, 2190.

11 S.E. Helaly, C. Richter, B. Thongbai, K.D. Hyde and M. Stadler, *Tetrahedron Lett*., 2016, **57**, 5911–5913.

12 C. Chepkirui, K.T. Yuyama, L.A. Wanga, C. Decock, J.C. Matasyoh, W.-R. Abraham and M. Stadler, *J. Nat. Prod.*, 2018, **81**, 778–784.
13 K. Yuyama, L. Wendt, F. Surup, R. Kretz, C. Chepkirui, K. Wittstein, C. Boonlarppradab, S. Wongkanoun, J. Luangsa-ard, M. Stadler and W.R. Abraham, *Biomolecules*, 2018, **8**, DOI:10.3390/biom8040129.
